# Evaluation of Pesticide Residue Dynamics in Lettuce, Onion, Leek, Carrot and Parsley

**DOI:** 10.3390/foods9050680

**Published:** 2020-05-25

**Authors:** Tereza Horská, František Kocourek, Jitka Stará, Kamil Holý, Petr Mráz, František Krátký, Vladimír Kocourek, Jana Hajšlová

**Affiliations:** 1 Crop Research Institute, Division of Crop Protection and Plant Health, Drnovska 507, 161 06 Prague, Czech Republic; tereza.horska@vurv.cz (T.H.); kocourek@vurv.cz (F.K.); stara@vurv.cz (J.S.); holy@vurv.cz (K.H.); 2 Department of Food Analysis and Nutrition, University of Chemistry and Technology, Technicka 3, 166 28 Prague, Czech Republic; petr.mraz@vscht.cz (P.M.); frantisek.kratky@vscht.cz (F.K.); vladimir.kocourek@vscht.cz (V.K.)

**Keywords:** pesticide residues, low-residue production, zero-residue production, half-lives, pre-harvest interval, lettuce, onion, leek, carrot, parsley

## Abstract

The dynamics of 32 active substances contained in pesticide formulations (15 fungicides and 17 insecticides) were analyzed in iceberg lettuce, onion, leek, carrot, and parsley. Pesticide residues were monitored from the time of application until harvest. In total, 114 mathematical models of residue dissipation were developed using a first-order kinetic equation. Based on these models, it was possible to predict the action pre-harvest interval (the time between the last pesticide application and crop harvest) needed to attain a targeted action threshold (value significantly lower than the maximum limit) for low-residue vegetable production. In addition, it was possible to determine an action pre-harvest interval based on an action threshold of 0.01 mg kg^−1^ to produce vegetables intended for zero-residue production. The highest amount of pesticide residues were found in carrot and parsley leaves several days after treatment, and pesticide dissipation was generally slow. Lower amounts were found in leeks and lettuce, but pesticide dissipation was faster in lettuce. According to our findings, it seems feasible to apply reduced pesticide amounts to stay below unwanted residue levels. However, understanding the effectivity of reduced pesticide application for controlling relevant pest organisms requires further research.

## 1. Introduction

Conventional farming and integrated pest management (IPM) regimes relate to the utilization of pesticides for the protection of agricultural crops against diseases and pests. However, pesticides are potentially toxic to humans and can have both acute and chronic health effects, depending on the quantity and ways in which a person is exposed [1]. Hence, controlled use of agricultural pesticides is important, modelling pesticide distribution in crops as a tool in limiting the excessive use of pesticides [2]. Maximum residue limits (MRLs) for pesticide/commodity combinations have been established by the European Union in the Regulation of European Commission [3] as the highest level of pesticide residues that are legally tolerated in food or feed. MRLs are based on Good Agricultural Practice (GAP) data and must meet requirements for pesticide registration. A uniform MRL as low as 0.01 mg·kg^−1^ has been established by Commission Directives [4,5] for any pesticide residue in baby foods and processed cereal-based foods [6]. To meet the legal limits for pesticide residues in various food crops at a given harvest time, a pre-harvest interval (PHI) is officially established for particular pesticides defined as the time between the last pesticide application and the harvest of the treated crop. This value is based on FAO (Food and Agriculture Organization of the United Nations) recommendation [7] and is usually fixed at a country level [8]. The aim of “zero-residue” vegetable production (“pesticide residue free production”) is achieving pesticide residues in respective crops as low as 0.01 mg·kg^−1^ (or even lower). This limit corresponds to the limit used for baby food production [4]. Adopting MRLs and PHIs helps to keep residue-related health risks below an “acceptable risk level”.

The most recent EU report summarizes the study of pesticide residue contamination of food carried out in the European Union (EU) Member States, Iceland, and Norway [9]. It summarizes the results of both the 2017 EU-coordinated control program (EUCP) and the national control programs (NP). To allow the assessment of representative consumer exposure to pesticide residues by food commodity, the same pattern of commodities is monitored for the presence of pesticide residues in 3-year cycles in the same countries. Regarding the 2017 EUCP, results were compared with the ones of 2014 for those commodities sampled in both years. The results of this showed some improvement in the overall situation in comparison with the results of 2014. No quantifiable residues were reported in 54.1% and 53.6% of the samples in 2017 and 2014, respectively. Quantified residues at or below the MRLs were reported in 41.8% and 43.4% of the samples in 2017 and 2014, respectively [9]. Overall, 95.9% of the samples analyzed in 2017 fell within the legal limits; nevertheless, 41.8% of the samples contained quantified residues at or below the MRLs. Nevertheless, the dietary risk assessment indicated that, for the samples analyzed, the probability of European citizens being exposed to pesticide residue levels that could lead to negative health outcomes is relatively low [9].

However, supermarkets, and thus also food crop producers, are continuously under pressure due to consumer concern about the health risks associated with the potential occurrence of pesticide residues in their diet. When seeking to rationalize pesticide use, both government [10] and supermarkets have tended to make the implicit assumption that any rationalization in this field is primarily an issue of decreasing the quantity of pesticides used, coupled with the banning of certain persistent or highly toxic substances [11,12]. Some retail chains apply for the assessment of contamination of products, mainly fruits and vegetables, originating from low-residue production. So called “action thresholds” declare the highest acceptable concentration of pesticide residues in the product corresponding to a percentage of the respective MRL, e.g., 25%. The required action thresholds commonly range from 25% to 75% MRL depending on the retail chain. In all cases, the action thresholds are fairly below the MRL. Achieving action thresholds for low-residue production or zero-residue production is based on a setting of an action pre-harvest interval (APHI), which is the minimal time between the last pesticide application and crop harvest within which the required drop in pesticide residue levels occurs. The knowledge of APHIs, which is necessary for setting relevant pesticide treatment regimes in low-residue and/or zero-residue production, needs the development of dissipation models of the active substances in pesticide preparations, based on which calculations are performed. In this context, information on the dissipation kinetics of pesticide residues in food crops and other plants is an additional key aspect of current risk and impact assessment practices. Considering more than 740 published studies, Fantke and Juraske [13] identified nine different calculation models, corresponding residual pesticide concentration curves *C_t_* and related half-lives *t_1/2_*. The authors concluded that pesticide dissipation in plants generally follows first-order kinetics, although some exceptions exist. Currently, experimental data are not available for all relevant pesticide−plant combinations; nevertheless, Fantke et al. [14] created a list of corrected geometric means of dissipation half-lives for 333 pesticides applied to an average plant under reference air temperature (20 °C). Half-lives *t_1/2_*_ref,i_ were calculated using model II based on dissipation data obtained for 346 pesticide residues in 183 treated plant species [13,14]. Model II also provides estimates for the influence of specific plants, which can be used to correct *t_1/2_*_ref,i_ for pesticides applied to a particular crop under given study conditions and temperature. Model III was designed to estimate dissipation half-lives from physico-chemical properties of individual pesticides of 14 substance classes for which no reference half-lives are available, i.e., they are not listed in *t_1/2_*_ref,i_ [14]. The model III is more uncertain as it tries to predict half-lives for chemicals not included in the fitting dataset.

Pesticide residues were analyzed in Brassica vegetables with application until harvest in order to establish mathematical models of residue dissipation and forecast the action pre-harvest interval for a given action threshold as a percentage of MRL for low-residue production in Brassica vegetables [15]. This study describes the dissipation of 22 active substances of pesticide formulations applied on Chinese cabbage, head cabbage, and cauliflower.

Experimental studies usually present aggregated dissipation data estimated from measuring the changes of the overall residual pesticide concentration in the respective plant over time. However, the overall dissipation of residues involves several processes. Jacobsen et al. [2] showed that degradation of a pesticide molecule is, in many cases, the most relevant dissipation component, followed by growth dilution and volatilization. Dissipation rate and half-lives of pesticides vary according to vegetable species and depend on plant characteristics, environmental conditions, and substance physico-chemical properties [7]. Hence, the models of pesticide dissipation are applicable in regions with similar climatic conditions and under comparable conditions of vegetable growing [2].

The main objective of the study was to evaluate dynamics of pesticide residues in iceberg lettuce, onion, leek, carrot, and parsley, and, based on generated data, to suggest a procedure enabling targeted (low) levels of pesticide residues in respective vegetable at harvest. The specific objectives of this work were (1) to quantify the dissipation rates of pesticide residues in tested vegetables, (2) to determine the APHI_25_ and APHI_0.01_ that would guarantee achieving “action thresholds” of 25% MRL and 0.01 mg residue kg^−1^, respectively, for a low-residue and zero-residue production of tested vegetables, and (3) to critically assess differences in the dissipation rates and dissipation half-lives of particular pesticide residues in tested vegetable species and compare the results with available literature data.

## 2. Materials and Methods

Pesticide residues were analyzed in samples collected from semi-field experiments on iceberg lettuce (*Lactuca sativa* L.), leek (*Allium ampeloprasum* L.), carrot (*Daucus carota* L. subsp. sativus (Hoffm.) Arcang), and parsley (*Petroselinum crispum* (Mill.) Fuss) at the Crop Research Institute in Prague (GPS: 50.0864428N, 14.2985553E, soil type: illimerized luvisol, soil pH: 6.8) and on onion (*Allium cepa* L.) at the Czech University of Life Sciences Prague (GPS: 50.1267258N, 14.3770625E, soil type: black earth, pH: 7.3). The specific information of the geographic coordinates, soil type and soil pH are presented according to current reporting recommendations [16]. The studied vegetables were usually treated with pesticides registered for the control of vegetable diseases and pests in the Czech Republic. However, some additional pesticides permitted in neighboring countries were also selected for the case of their future use in the Czech Republic. Iceberg lettuce and both root vegetables were planted in a single season, while onion and leek were cultivated in two consecutive years (Table 1). Cultivation technologies (row and in-row plant spacing, fertilization) respected general field practices. The same pesticide application and sampling methodology were used for all tested vegetable crops. The average air temperature (min/max range) from the first chemical treatment until the last harvest was 20 °C (13.8/26.8 °C) in lettuce 2011; 18.3 °C (13.7/29.7 °C) and 17.3 °C (12.8/24.7 °C) in onion in 2012 and 2013, respectively; 9 °C (1.7/13.9 °C) in leek in 2008; 10.5 °C (1.7/19.3 °C) in leek as well as in carrot in 2009; and 12 °C (6.4/15.2 °C) in parsley in 2010. The crops were cultivated in three plots (A–C); three replications per plot were set up in a randomized complete block design. Each plot was treated with three different pesticide mixtures (Supplementary Table S1 online) on three different dates (Table 1). Thus, pesticide mixture 1 was sprayed in plot A on the first date, in plot B on the second date and in plot C on the third date of experiment. Pesticides in each mixture are marked with a superscript in Supplementary Table S1 online. The pesticide mixtures were different for each crop. The mixture composition depended on the efficacy of the pesticides in each crop. Pesticides were applied from the dates selected based on plant phenophase and the estimated harvest date. Plant stages at the time of application of pesticides were BBCH 42, 45, 45, 45, and 45 for iceberg lettuce, leek, carrot, parsley, and onion, respectively [17]. All the pesticides were administered at maximum label application rates. In all variants, 500 L·ha^−1^ spraying with 0.1 L·ha^−1^ Silwet was used. Sample collection started three days after the last spraying. In each plot, the samples were collected on four different dates; thus, twelve samples of each vegetable were analyzed per year except for onion, where only three sample collections were performed in 2012. Crops, planting dates, varieties, pesticide application dates, and harvest dates are listed in Table 1.

Analyses of pesticide residues were performed by the testing laboratory of the University of Chemistry and Technology accredited according to the EN ISO/IEC 17025 standard. The standardized analytical method used in this study is based on the EN 12393-2 standard (Foods of plant origin-Multiresidue methods for the determination of pesticide residues by GC or LC-MS/MS-Part 2: Methods for extraction and clean-up) and EN 12393-2 (Foods of plant origin-Multiresidue methods for the determination of pesticide residues by GC or LC-MS/MS-Part 3: Determination and confirmatory tests).

Approximately 1 kg of representative vegetable samples from field experiments were delivered to the laboratory at the University of Chemistry and Technology in Prague. The edible part of the crop and the top leaves were separated and homogenized using a Retsch GM200 blender (Retsch GmbH, Haan, Germany) and stored in a freezer (−18 °C) until analysis. An ISO 17025 [18] accredited multiresidue method based on QuEChERS extraction followed by high-performance liquid chromatography coupled with tandem mass spectrometry (HPLC-MS/MS) was employed, encompassing most of the examined pesticides (abamectin, acetamiprid, azoxystrobin, boscalid, chlorantraniliprole, chlorpyrifos, cymoxanil, cyprodinil, cypermethrin, deltamethrin, difenoconazole, dimethoate, dimethomorph, fludioxonil, fluoxastrobin, indoxacarb, mandipropamid, metalaxyl-M, methoxyfenozide, pirimicarb, propamocarb-hydrochloride, prothioconazole, pyraclostrobin, pyridaben, spinosad, tebuconazole, thiacloprid, thiamethoxam); for pymetrozine, a single residue method with pH adjustment of the sample was performed. Sample preparation and HPLC-MS/MS analysis were described in detail in our previous study [19], and the multiple reaction monitoring (MRM) conditions optimized for the pesticides evaluated in this study are summarized in Supplementary Table S2 online. These analytical procedures were introduced earlier by Ticha et al. [20] and subsequent validation protocol as well as the Internal Quality Control (IQC) measures followed document SANTE/11945/2015 (latest consolidated version SANTE/12682/2019) “Method Validation & Quality Control Procedures for Pesticide Residues Analysis in Food & Feed” [21], obligatorily used by the EU official control laboratories. MS measurement parameters of the optimized method are given in Table S2 (LC-based) and Table S3 (GC-based, confirmatory for GC amenable pesticides) in Supplementary Materials. The performance characteristics for all pesticides involved in this study are summarized in Supplementary Table S4 online. The mean recovery, repeatability standard deviation, and limit of quantitation (LOQ) were evaluated for each pesticide. This means that the entire analytical procedure (started by the handling of the analytical portion) is represented by the metrological characteristics given in Table S4. The performance of the accredited laboratory was verified (external quality control, QC) through participation of the laboratory in regular proficiency tests: (i) Food Analysis Performance Assessment Scheme (FAPAS^®^) and (ii) European Commission’s Proficiency Testing Program (EU–PT).

The following first-order kinetic equation was used to characterize the dissipation rate of active substances of the pesticides in crop products:

*C_t_* = *C*_0_ × exp(−*k*^*diss*^ × *t*) (1)

where *C_t_* is the residual pesticide concentration (mg·kg^−1^) at time *t* (days) after application; *C_0_* is the initial pesticide concentration; and *k^diss^* is the pesticide dissipation rate constant (day^−1^). ANCOVA was used to analyze the effect of the year and time after application on the residual pesticide concentration. If there was no significant difference between the years, the data from two years were combined. The corresponding dissipation half-life equation is:

*t*_1/2_ = ln(2)/*k*^*diss*^ (2)

where *t_1/2_* is the pesticide dissipation half-life (days), which was used for all pesticides with significant models (*R*^2^ > 0.5). The model parameters of *C_0_* and *k^diss^* were calculated from all experimentally determined residue values of each active pesticide substance in the products (*C_t_*) and the corresponding number of days after pesticide application (*t*). Calculations were performed by Kruskal–Wallis test in the XLSTAT 2009 program (Addinsoft, New York, NY, USA). Our *t_1/2_* values were compared with corrected geometric means of estimated half-lives applied to specific plants in each set of production conditions and temperature using model II [14]. Two active substances (fluoxastrobin [22] and prothioconazole [23]) were not involved in the pesticide list needed for the *t_1/2_* calculation according to model II, so we used model III, which Fantke et al. [14] designed to predict the half-lives of individual pesticides for which no reference half-lives are available.

Maximum residue limits were obtained in EU Pesticides database [24]. Because the MRL of active substances used for carrot and parsley roots was the same, the missing MRL for carrot leaves was replaced by the MRL of parsley leaves. MRLs for carrot leaves are not established because carrot is grown almost exclusively for the root. However, raw young carrot leaves have recently been used not only as food for animals but also to enrich dishes such as salads or soups. “Action thresholds” for the 25% MRL (APHI_25_) and 0.01 mg kg^−1^ limit (APHI_0.01_) were established for 31 active substances in the pesticide formulations. To calculate the APHI, the following equation can be used:

*t* = (ln *C_t_* − ln *C_0_*)/*k^diss^* (3)

where *C_t_* is the residual pesticide concentration in mg·kg^−1^ at time *t* (days) after application; *C_0_* is the initial pesticide concentration; and *k^diss^* is the pesticide dissipation rate constant (day^−1^). For zero-residue production, *C_t_* = 0.01 mg·kg^−1^; for low-residue production, *C_t_* corresponds to a given percentage of the MRL (e.g., 25% MRL). The APHIs calculated from the pesticide dissipation models were extended by one-third based on a confidence interval of the model for active substances with the highest variability of dissipation rate to increase the reliability of APHIs, i.e.,

APHI = *t* + (1/3*t*) (4)

The parameters *C_0_* and *k^diss^* differ according to the active pesticide substance and the agricultural commodity. When the calculated APHI prolonged by one-third was shorter than the obligatory PHI according to a list of registered products [25], the PHI was used.

## 3. Results

### 3.1. Pesticide Dissipation Models, Dissipation Half-Lives and “Action Pre-Harvest Intervals”

The residues of 21 active substances were determined in lettuce, 29 in onion, 13 in leek, 9 in carrot, and 10 in parsley. The mathematical models could not be established for three pesticide active substances in onion, one on leek, two in carrot roots, and five in parsley roots because the concentration of residues in vegetables rapidly decreased after the application to very low levels. The parameters of 114 pesticide dissipation model equations and dissipation half-lives are shown in Supplementary Table S5A–E online. For seven active substances on onion, five on leek and parsley, and two on carrot, the calculation of model parameters yielded non-significant values (*R*^2^ < 0.5) due to a high data variability. The results of residue analyses (mg·kg^−1^) corresponding to the number of days in the obligatory PHI are shown in the following tables (Table 2, Table 3, Table 4, Table 5 and Table 6), as are APHI_25_ and APHI_0.01_ calculated for low-residue and zero-residue vegetable production, respectively. In cases where the models were not established because of rapid residue dissipation, APHI_25_ and APHI_0.01_ were identical to the PHI. Otherwise, the calculated APHI_25_ and APHI_0.01_ were used when their values were lower than the obligatory PHI. In several cases in which the MRL was very low (0.01–0.03 mg·kg^−1^), the 25% MRL used to calculate APHI_25_ was lower than 0.01 mg·kg^−1^, so the calculated APHI_0.01_ was shorter. At present, zero-residue production requires a pesticide residue limit of 0.01 mg·kg^−1^, so that in the cases in which the calculated APHI_25_ is longer, APHI_0.01_ must be used instead of APHI_25_.

**Figure 1:**
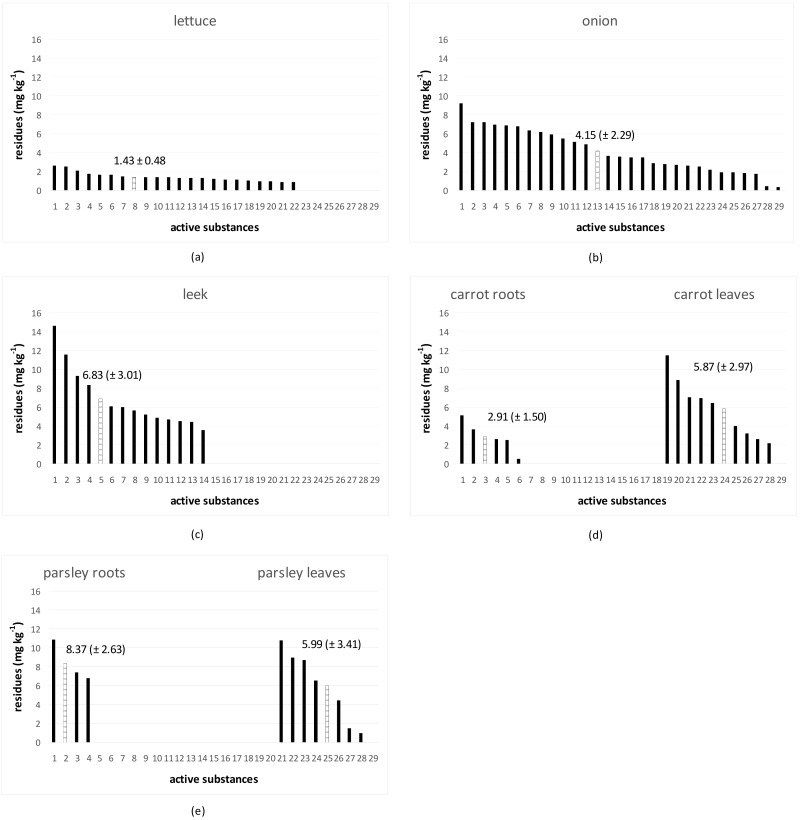
Half-lives of active substances (black columns) in decreasing order (**a**) lettuce; (**b**) onion; (**c**) leek; (**d**) carrot; (**e**) parsley. Mean half-live ± SD (hatched column). Units on the x axis represent the active substances listed in Supplementary Table S6 online.

The half-lives of active substances were shortest in iceberg lettuce where the mean half-life of all tested active substances was 1.43 (± 0.48), (Figure 1a). In onion, the mean half-life of all tested active substances was 4.15 (± 2.29), indicating the third fastest dissipation from all crops (Figure 1b). Leek belonged to the crops with higher half-life values. The mean half-life of all tested active substances was 6.83 (± 3.01) (Figure 1c). The mean half-lives of all tested active substances in carrot roots and leaves were 2.91 (± 1.50) and 5.87 (± 2.97), respectively (Figure 1d). The half-lives of active substances in carrot roots belonged to the shorter ones, whilst the half-lives of active substances in leaves exceeded six days in five cases. The mean half-lives of all tested active substances in parsley roots and leaves were 8.37 (± 2.63) and 5.99 (± 2.63), respectively (Figure 1e). The mean half-life of active substances in parsley root was the longest, however the concentration of pesticides detected in parsley roots was lower than 0.09 mg·kg^−1^ (Supplementary Table S7 online) and the dissipation of other five tested substances was very fast, the determination of their half-lives was impossible. The half-lives of active substances in parsley leaves were comparable to those determined for carrot leaves.

#### 3.1.1. Iceberg Lettuce

In iceberg lettuce, 21 significant (*R*^2^ = 0.761−1.000) pesticide dissipation models were generated (Supplementary Table S5A online). The half-lives in iceberg lettuce were in range 0.86 days (dimethomorph) to 2.65 days (thiamethoxam), as shown in Table S5A. The application of all active substances before PHI will allow residues below MRL (see slope model in Table 2). Dissipation models produce APHI_25_ values lower or equal to the PHI. In the case of chlorpyrifos, iprodione and tebuconazole, the PHI was extended for a few days (Table 2). The calculated APHI_0.01_ times for acetamiprid, chlorpyrifos, iprodione, lambda-cyhalothrin, mandipropamid, pirimicarb, pymetrozine, and thiacloprid were two to six days longer than the PHI. In tebuconazole, the limit for baby food exceeded nine days.

#### 3.1.2. Onion

In onion, 29 active substances were analyzed, 27 of which were sprayed in two seasons, 2012 and 2013, and chlorantraniliprole and propamocarb-hydrochloride were tested in the second year only (Supplementary Table S1 online). In total, 52 significant (*R*^2^ = 0.500–1.000) and 7 non-significant models were established (Supplementary Table S5B online). In 12 active substances, significant models were established from years as well as from both years analyzed together and only difenoconazole and fludioxonil showed differences between the years. Therefore they were evaluated in both years separately. No models were generated for three active substances (abamectin, deltamethrin, and lambda-cyhalothrin) in onion due to the low concentration of their residues in terms of harvest in both years. Similarly, no models were established for cymoxanil, prothioconazole and thiamethoxam in one year (Table S5B). Residue half-lives in onion ranged from 0.36 days (cymoxanil 2012) to 9.24 days (boscalid 2012), as shown in Table S5B. The application of active substances before the PHI will allow residues below MRL, except for iprodione and methoxyfenozide, which slightly exceeded the MRL in one year (see slope model in Table 3). In 9 active substances of pesticides, the calculated APHI_25_ was longer than the APHI_0.01_ due to a low MRL of 0.01–0.02 mg·kg^−1^. In such cases, the APHI_25_ was not relevant. For example, the 25% MRL “action threshold” for acetamiprid in 2013 was 13 days and the APHI_0.01_ was 8 days (Table 3). In most cases, the APHI_25_ was shorter or the same as the PHI. In the case of tebuconazole in 2013, the APHI_25_ was longer than the PHI for 5 days (Table 3). The calculated APHI_0.01_ values for 10 substances were longer than the PHI. In some cases, the difference in APHI_0.01_ and PHI was even more than 20 days (boscalid 2012, tebuconazole 2013).

#### 3.1.3. Leek

In leek, 13 active substances were evaluated; seven of them were sprayed in two seasons, 2008 and 2009 (Supplementary Table S1 online). Seventeen significant (*R*^2^ = 0.568–0.939) and five non-significant pesticide dissipation models were established. In azoxystrobin and thiacloprid, significant models were established from years as well as from both years analyzed together. As the data obtained for deltamethrin showed a difference between the years, evaluation of this insecticide was performed separately in both years No model was generated for abamectin due to its rapid dissipation in leek (Supplementary Table S5C online). The ranges of the half-lives in leek were 2.16 days (thiacloprid 2009) to 14.65 days (deltamethrin 2008) (Table S5C). The application of these active substances before the PHI did not exceed the MRL, except for acetamiprid, chlorpyrifos, and thiamethoxam (see slope model in Table 4). For acetamiprid, chlorpyrifos, and thiamethoxam, the calculated APHI_25_ was longer than the APHI_0.01_ due to the low MRL of 0.01 mg kg^−1^. In such cases, the APHI_25_ was not relevant. For pyridaben and tebuconazole, the APHI_25_ was longer than the PHI by 18 and 8 days, respectively. The calculated APHI_0.01_ time was longer for all tested active substances than the PHI. The longest extension time of PHI was 34 days to 55 days for azoxystrobin 2008 (Table 4).

#### 3.1.4. Carrot

In carrot, residues of nine active substances were evaluated in the root and leaves separately. Five (root; *R*^2^ = 0.516–1.000) and nine (leaves; *R*^2^ = 0.713–0.986) significant pesticide dissipation models were established. No models were generated for two active substances (deltamethrin and lambda-cyhalothrin) due to their rapid dissipation in carrot roots (Supplementary Table S5D online). The half-lives of pesticide residues in carrot roots and leaves were in the range of 0.56 days (acetamiprid) to 5.13 days (cypermethrin) and 2.14 days (spinosad) to 11.50 days (cypermethrin), respectively, as shown in Table S5D. The application of active substances before the PHI will allow residue below MRL except for acetamiprid and tebuconazole in leaves (see slope model in Table 5). In carrot roots, dissipation models produce APHI_25_ and APHI_0.01_ values that are equal to the PHI, except for azoxystrobin, where the APHI_0.01_ time exceeded the PHI by 8 days. In carrot leaves, the calculated APHI_25_ time exceeded the PHI in cypermethrin and tebuconazole by more than 1.5 and 3 times, respectively. The APHI_0.01_ limits prolonged the PHI in all active substances by a minimum of 10 days in spinosad and a maximum of 111 days in cypermethrin (Table 5).

#### 3.1.5. Parsley

In parsley, residues of ten active substances were evaluated in the root and leaves separately. Three (root; *R*^2^ = 0.532–0.778) and seven (leaves; *R*^2^ = 0.707–0.985) significant pesticide dissipation models were established. No models were generated for five active substances (cypermethrin, deltamethrin, lambda-cyhalothrin, metalaxyl-M, and pirimicarb) due to their rapid dissipation in parsley roots (Supplementary Table S5E online). The half-lives in parsley roots were in the range 6.81 days (thiacloprid) to 10.88 days (tebuconazole). In parsley leaves, the half-lives ranged from 0.97 days (azoxystrobin) to 10.89 days (lambda-cyhalothrin), as shown in Table S5E. The application of all active substances before PHI will allow residue below MRL. In parsley roots and leaves, dissipation models produce APHI_25_ values that are equal to the PHI. In parsley roots, the calculated APHI_0.01_ time was, for azoxystrobin and tebuconazole, more than two and six times longer than the PHI, respectively. In parsley leaves, the limit for zero-residue production was highly exceeded in five active substances by a minimum of 23 days in spinosad and a maximum of 52 days in cypermethrin (Table 6).

### 3.2. Dissipation Course of Active Substances from Application to Harvest

**Figure 2:**
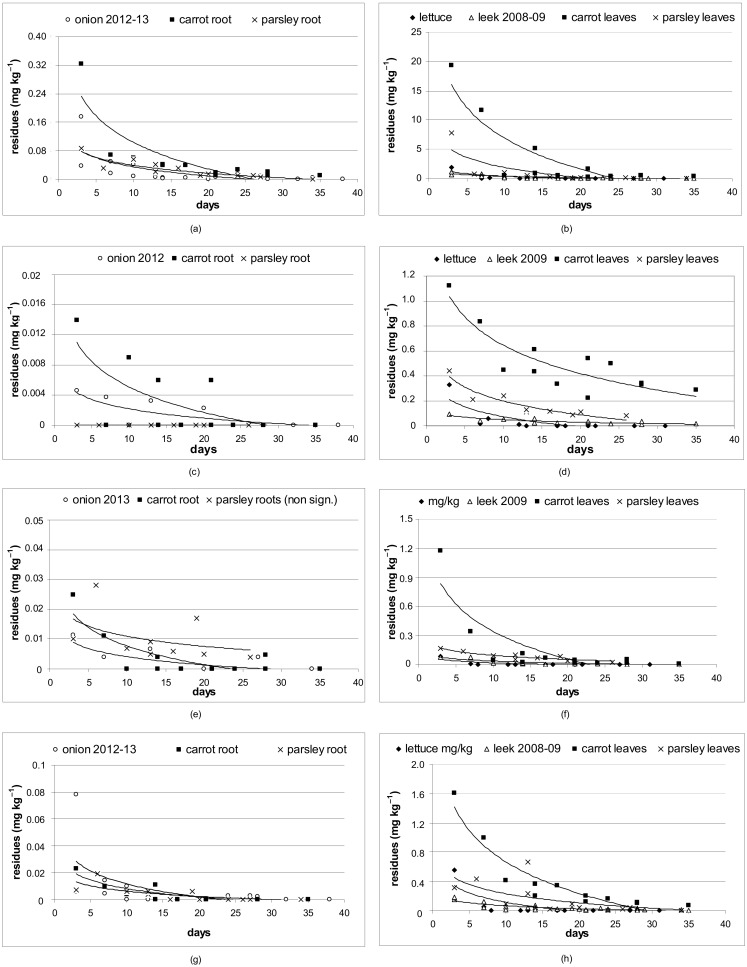
Dissipation of four active substances in vegetable species. (**a**) azoxystrobin on bulb and roots; (**b**) azoxystrobin on aboveground parts of crops; (**c**) cypermethrin on bulb and roots; (**d**) cypermethrin on aboveground parts of crops; (**e**) spinosad on bulb and roots; (**f**) spinosad on aboveground parts of crops; (**g**) thiacloprid on bulb and roots (**h**) thiacloprid on aboveground parts of crops.

Eight of the thirty-two active substances were sprayed on all experimental vegetable species. (Table S7). One fungicide (azoxystrobin) and three insecticides (cypermethrin, spinosad and thiacloprid) were selected to demonstrate differences in pesticide dissipation behavior (Figure 2). Higher levels of residues of four active substances in the first term after application were found on lettuce, leek, and carrot and parsley leaves. The highest levels were observed for azoxystrobin (20 mg kg^−1^) and for tested insecticides (from 1.1 to 1.6 mg kg^−1^). Low levels of residues of azoxystrobin (0.3 mg kg^−1^) and tested insecticides (ranging from 0.02 to 0.014 mg kg^−1^) were observed in the first term after application in onion. Less residues of active substances were found in the underground part of crops than in the above parts. The highest number of residues were found in carrot leaves, in descending order followed by parsley leaves, lettuce, leek, carrot root, parsley root, and onion (Figure 2). The highest levels of residues were found for azoxystrobin, followed by cypermethrin, and the lowest values of residues were observed for thiacloprid and spinosad.

## 4. Discussion

In the tested vegetables, the pesticide dissipation rate *k^diss^* and the pesticide dissipation half-life *t_1/2_* varied depending on the active substances and vegetable species. The fastest pesticide dissipation occurred overall in lettuce (Table S5A; Figure 1a). Our results corresponded with the findings of Song et al. [27], who compared dissipation curves and half-lives of six pesticides on six leafy vegetables planted in field trails. The dissipation of dimethoate, chlorpyrifos, beta-cypermethrin and deltamethrin in leaf lettuce expressed in *k^diss^* and *t_1/2_* was in the range of −0.793 to −0.176 and 0.87 to 3.94 days, respectively.

The dissipation half-lives of the active substances were compared with the predicted geometric mean of the dissipation half-lives at 20 °C (*t_1/2_*_ref,i_) and the corrected *t_1/2_*_ref,i_ of pesticides applied to a specific vegetable under a given average temperature (*t_1/2_*_plant, active subst._) calculated according to model II [14]. The results are given in Table S7. In many cases, the corrected *t_1/2_*_plant, active subst_ of the active substances correspond more to our results; e.g., the corrected *t_1/2_* in lettuce and onion were shorter than *t_1/2_*_ref,i_, whereas in leek, the corrected half-lives were prolonged for several days (Table S7). Two active substances, fluoxastrobin and prothioconazole, sprayed on onion did not appear in the pesticide list needed for *t_1/2_* calculation according to model II. We compared our results with *t_1/2_*_ref,i_ and *t_1/2_*_plant, active subst._ computed according to model III [14]. Although the calculation according to model III has its limitations, such as higher uncertainty compared to half-lives based on model II [14], the results correlated with our values (Table S7). Computed predicted half-lives (*t_1/2_*_ref,i_; *t_1/2_*_plant, active subst._) of fluoxastrobin 2012–13 (6.14; 6.73 days) and prothioconazole 2013 (7.19; 8.14 days) corresponded to our results of 7.20 and 6.78 days, respectively.

Eight of the thirty-two active substances were sprayed on all vegetable species. The results showed differences in concentrations of active substances in underground and aboveground parts of the plants after foliar application of the pesticides. The initial deposition of the active substance in aboveground part could be influenced by the foliar surface area, which can be defined as vegetation cover (VC), e.g., the proportion of soil area covered by leaves [8]. Leaves of carrot and parsley are overlapping, while the compact shape of iceberg lettuce head and leek could allow less deposition of the active substance. The role of foliar area in the initial deposition of active substances has been mentioned, e.g., by Lu et al. [28] and Song et al. [27]. Two fungicides (azoxystrobin, tebuconazole) and three insecticides (cypermethrin, spinosad, thiacloprid) were selected to demonstrate differences in pesticide dissipation.

The systemic fungicide azoxystrobin had robust models for all vegetables and its parts. The dissipation rate of azoxystrobin was as follows (ranked from the fastest dissipating): lettuce, parsley leaves, carrot root, onion, carrot leaves, leek, and parsley root. The coefficient *k^diss^* ranged from −0.725 to −0.093, and *t_1/2_* ranged from 0.96 to 7.42 days. Azoxystrobin belonged to eight active substances most often detected in vegetables and mushrooms obtained during official inspection of pesticide residues in Czech supermarkets in 2018 [29]. Similarly, azoxystrobin was detected in eight vegetable species from southeastern Poland but never exceeded the MRL [30]. According to our results, azoxystrobin never exceeded the MRL and in all tested vegetables produced APHI_25_ values that were equal to or lower than the PHI, but it was not suitable for zero-residue production, except for lettuce.

The second fungicide, tebuconazole, had significant effects on all vegetables except carrot root and parsley leaves. The dissipation rate of this systemic triazole fungicide was as follows (ranked from the fastest): lettuce, leek, onion, carrot leaves, and parsley root. The dissipation constant *k^diss^* and *t_1/2_* ranged from −0.523 to −0.064 and 1.32 to 10.88 days, respectively. Tebuconazole is not allowed on any of the tested vegetables in the Czech Republic, and our results confirm that it is not suitable for low-residue or zero-residue production.

Spinosad, a fermentation product of the actinomycete bacterium *Saccharopolyspora spinosa*, dissipated in descending order: lettuce, carrot leaves, carrot roots, onion, parsley leaves, and leek. In parsley roots, the dissipation model was not significant. Based on the *k^diss^* (−0.614 to −0.132) and *t_1/2_* (1.13 to 5.23 days) values, spinosad is a rapidly dissipating substance. In the Czech Republic, spinosad is permitted in lettuce (PHI 14 days), onion, and leek (PHI 7 days). In carrot and parsley, we used a longer PHI of 14 days. In all tested vegetables, spinosad was suitable for low-residue production with APHI_25_. In lettuce, carrot root and onion, production with less than 0.01 mg kg^−1^ spinosad was feasible. Sikorska-Zimny et al. [31] calculated the half-life of spinosad in onion, carrot and cabbage and found the values to be 5.2, 3.6 and 2.9 days, respectively. Our calculated half-lives of spinosad in onion (3.58 days), carrot root (2.55 days), and cabbage (3.85 days; data shown in Kocourek et al. [15]) confirm the fast dissipation process of this substance.

Similarly, thiacloprid, a neonicotinoid insecticide, dissipated fast in almost all tested vegetables (ranked from the fastest): lettuce, onion, carrot root, leek, carrot leaves, and parsley roots, with *k^diss^* values of −0.660 to −0.102 and *t_1/2_* values of 1.05 to 6.81. The parsley leaf data did not enable the generation of any significant model. In the Czech Republic, thiacloprid is permitted in lettuce, carrot, and parsley with a PHI of seven days. In our study, we used the longest PHI of 21 days for leek and onion, as recommended for other vegetable species. In onion, the 21-day PHI was sufficient for 25% MRL as well as for zero-residue production. In the case of leek, a 21-day PHI was suitable for 25% MRL as well as for zero-residue production in one year only. In 2008, the calculated APHI_25_ and APHI_0.01_ prolonged the PHI for 6 and 18 days, respectively.

Cypermethrin, a synthetic pyrethroid, dissipated in descending order: lettuce, carrot root, onion, parsley leaves, leek, and carrot leaves, with *k^diss^* values of −0.507 to −0.051 and *t_1/2_* values of 1.37 to 11.53. In parsley roots, cypermethrin was below the detection limit. Its dissipation strongly depended on the vegetable species. For example, no cypermethrin residues were detected in carrot roots after foliar application or later [32]. On the other hand, Yuan et al. [33] reported a high concentration of cypermethrin in some vegetable samples from supermarkets, where residue levels varied in different vegetables, high levels being found in radish and cauliflower. Cypermethrin was the insecticide observed frequently at concentrations above the MRL in leafy vegetables [34]. Cypermethrin is the most used pesticide in vegetables, but the residue levels were always below the European MRL [35].

Two others tested pyrethroids (deltamethrin and lambda-cyhalothrin) dissipated according to vegetable species and their parts. Both were decomposed very fast in onion, carrot, and parsley roots without the ability to create a dissipation curve. Similarly, Riplay et al. [32] did not detect deltamethrin residues in onion from the first day of foliar application. In lettuce, the *k^diss^* coefficients −0.539 and −0.404 were obtained for deltamethrin and lambda-cyhalothrin, respectively. On the other hand, in leek, carrot, and parsley leaves, these two active substances dissipated very slowly and are thus not suitable for low-residue and zero-residue production.

Pesticides sprayed at the highest recommended dosages for vegetables in the Czech Republic dissipated under the MRL except for iprodione and methoxyfenozide (onion), acetamiprid, chlorpyrifos and thiamethoxam (leek) and tebuconazole (parsley leaves).

The MRLs of active substances in carrot and parsley roots can even be one thousand times lower than in leaves, e.g., the MRL of spinosad in parsley roots is 0.02 mg kg^−1^ and in parsley leaves is 60 mg kg^−1^ [24]. Our model for parsley roots was not robust enough, but the highest detected amount of spinosad was 0.03 mg kg^−1^ and in parsley leaves was 0.45 mg kg^−1^ three days after spraying. Similarly, the maximum level of spinosad detected in carrot roots and carrot leaves was 0.03 mg kg^−1^ and 1.18 mg kg^−1^ three days after spraying, respectively. The limit of 60 mg kg^−1^ appears to be very mild compared to the lettuce (10 mg kg^−1^) and leek (0.2 mg kg^−1^) MRLs. Leaves of parsley are often used raw in many dishes. High initial deposits of active substances in combination with mild MRLs allow PHI compliance.

Gonzáles-Rodríguez et al. [34] showed this problem in lettuce compared to other leafy vegetables. The MRLs for Swiss chards and spinaches can even be one hundred times lower than for lettuce because lettuce is highly sensitive to pests and needs successive applications of pesticides, consequently leaving a higher level of residues that are tolerated. Recently, there was a high decrease in the MRL of dimethoate in lettuce from 25 mg kg^−1^ (Reg. (EU) 2015/400) to 0.01 mg kg^−1^ (applicable from 31.7.2019; Reg. (EU) 2019/38) [24]. However, the MRLs of many active ingredients evaluated in lettuce were still much higher than those in leek (Table 2 and Table 4).

Models of pesticide dissipation in vegetables are suitable for the regulation of pesticide residues in harvested crops. In this paper, significant models were established for 114 pesticide/crop combinations. According to these models, it is possible to predict the APHI for the requested “action threshold” of residues in crops at the time of harvest. For pesticides with a very low incidence of residues in the short term after treatment, it is not necessary to prolong the PHI. Fast dissipation of residues was found for deltamethrin and lambda-cyhalothrin in onion, carrot roots, and parsley roots and for cypermethrin, metalaxyl-M and pirimicarb in parsley roots. Similarly, a very fast drop in residue levels was found for abamectin in onion and leek. For these pesticides, it is not necessary to establish APHI_25_ or APHI_0.01_ because the pre-harvest interval is achieved in any case. A strict limit for zero-residue production (0.01 mg kg^−1^) is achieved when pyrethroids are applied in root vegetables. In contrast, high deposits of pyrethroids in the leaves of carrot and parsley after treatment do not enable us to achieve this limit. Very low incidence of residues of several pesticides in the short term after treatment was also detected in cauliflower (beta-cyfluthrin) and head cabbage (acetamiprid, beta-cyfluthrin, cypermethrin, deltamethrin, lambda-cyhalothrin, pymetrozine, thiamethoxam) [15].

The difficulty of vegetable growing for low-residue or zero-residue production depends on the crop and pesticides used. The order of crops for which the established APHI_25_ or APHI_0.01_ are longer than the PHI corresponds to the order of crops descending from the lowest to the fastest dissipation of pesticides. For low-residue production, the order of crops is based on the number of pesticides with APHI_25_ values exceeding the PHIs from the total number of evaluated pesticides for which a significant model was established: leek (7/13), carrot leaves (2/9), iceberg lettuce (3/21), onion (4/29), parsley leaves (0/7), carrot roots (0/7), and parsley roots (0/8). In comparison, the number of pesticides with APHI_25_ values exceeding the PHIs was 7 of 20, 8 of 17, and 1 of 18 evaluated pesticides in Chinese cabbage, cauliflower, and head cabbage, respectively [15]. The growth of Chinese cabbage, leek and cauliflower for low-residue production is difficult, while the growth of iceberg lettuce, onion, head cabbage, parsley and carrot for roots seems to be easier. Growing of parsley and carrot for leaves in low-residue production is also achievable owing to the very mild MRLs discussed above. For zero-residue production, the order of crops is based on the number of pesticides with APHI_0.01_ values exceeding the PHIs from the total number of evaluated pesticides for which a significant model was established: leek (13/13), carrot leaves (9/9), parsley leaves (6/7), iceberg lettuce (9/21), onion (11/29), parsley roots (2/8), and carrot roots (1/8). In comparison, the number of pesticides with APHI_0.01_ values exceeding the PHIs were 19 of 20, 13 of 17 and 1 of 18 evaluated pesticides in Chinese cabbage, cauliflower, and head cabbage [15]. The growth of Chinese cabbage, leek, cauliflower, parsley, and carrot for leaves for zero-residue production is difficult, while the growth of head cabbage, iceberg lettuce, onion, parsley, and carrot roots is feasible.

According to the exceeding of the APHI_25_, it is possible to select the pesticides tebuconazole (onion, iceberg lettuce, carrot leaves, leek), chlorpyrifos (leek, iceberg lettuce), iprodione (onion, iceberg lettuce), cypermethrin (carrot leaves), methoxyfenozide (onion), azoxystrobin, pyridaben, thiacloprid, and thiamethoxam (leek) as unsuitable for low-residue production.

According to the exceeding of the APHI_0.01_, the number of “risky” pesticides is higher. Chlorpyrifos, acetamiprid, thiacloprid, and thiamethoxam were the insecticides that most often exceeded the APHI_0.01_ in vegetables. Except for acetamiprid, these pesticides were recently restricted by EU regulation [36,37,38]. Azoxystrobin, boscalid, iprodione, spinosad, pyridaben, propamocarb-hydrochloride, and tebuconazole were the fungicides that most often exceeded the APHI_0.01_ in vegetables. However, the limited number of pesticides available for the control of vegetables such as leek, Chinese cabbage or cauliflower did not enable the exclusion of these unsuitable pesticides from pest management.

The half-lives of pesticides set employed in this study for treatment of lettuce were lower than half-lives of those when used in onion, leek, and parsley and carrot roots, including the carrot leaves (Figure 1a). This means that dissipation rates of pesticides in lettuce were higher compared to other vegetable species (Supplementary Table S5 online). In this context it is rather surprising that relatively high levels of some fungicides (e.g., azoxystrobin) were found in samples of lettuce collected in a Spanish market, and in some of them the MRL was exceeded [34]. It is worthwhile to point out that MRLs for the same pesticides in other crops are in some cases lower than in lettuce [24]. For instance, the MRL for azoxystrobin in lettuce is 1.5-times higher than in onion and 15-times higher than in carrot root. The MRL for difenoconazole in lettuce is 10-times higher than in carrot and MRL for mandipropamid in lettuce is even 250-times higher than in onion [24]. MRLs for leafy vegetable as Swiss chard or spinach can be one hundred times lower than for lettuce [34]. The reason of such apparent discrepancies in MRLs setting is a high sensitivity of lettuce to pests and thus the need to apply more treatments, which, consequently, leave higher residues. However, in an earlier Spanish study, the residues of azoxystrobin in greenhouse lettuce harvested at PHI day were in all cases below the officially set MRL mentioned in the study. The results indicate that the time between application and harvest is at least as important as the application dose [8].

It is necessary to mention that high half-life values of some pesticides do not necessarily lead to occurrence of residues at harvest. For instance, thiamethoxam had in lettuce a relatively high level of half-live in comparison to onion (Table S5A,B). However, in lettuce the thiamethoxam level lowered at harvest below a limit of detection when PHI was kept [39].

The occurrence of pesticide residues in lettuce in a Spanish market was reported more often than in other vegetables [34] and may potentially pose a threat to consumer health. Interestingly, fungicides were most detected, especially in lettuce. Although concentrations of insecticides used to be lower, residues of cypermethrin, chlorpyrifos, difenoconazole, or lambda-cyhalothrin were found in 50% of the lettuce samples, while no detectable pesticide residues were present in potatoes and onions. In 20% of the lettuce samples, residues exceeded MRL; moreover, in some samples, this was the case for two or three pesticides [40].

Pesticide dissipation half-lives in plants are largely dependent not only on their characteristics (e.g., surface morphology) but also on environmental conditions [13]. Moreover, dissipation rate and half-lives of pesticides are variable for various vegetable species. Therefore, the mathematic models are suitable only for the vegetable species for which they were developed. One of the key factors affecting pesticide half-lives is temperature in the period between application and harvest, with higher temperatures resulting in higher dissipation rates and thus shorter half-lives. Generally, the PHI forecast according to developed models will be more reliable under conditions with higher temperatures. The application of the predictive models on vegetables grown in glasshouses is rather complicated as the temperature conditions are much higher than those for which they were developed; half-lives are fairly lower, what can be also documented by our earlier data comparing the fate of azoxystrobin under field and glasshouse conditions [8].

It is noteworthy that the developed models of pesticide dissipation for a forecast of PHI and incidence of residues in plant products before harvest are applicable in regions with similar climatic conditions for the growing of that vegetable. For expanded use of dissipation models developed in this study, parameters such as plant characteristics, substance properties and environmental conditions including temperature are summarized in regression models developed by Fantke et al. [14]. As an example, corrected values of half-live *t_1/2_*_ref,i_(day) and *t_1/2_*_plant, active subst._ (day) according to these regression models of Fantke et al. [14] are presented in Table S7.

Degradation is for many pesticides the most relevant dissipation process, followed by growth dilution [2]. Growth of plants, depending on water accessibility, is another limiting factor of this model’s reliability. A better forecast power can be expected when water access for vegetable is sufficient, regardless of whether it is due to irrigation or precipitation in the growing locality. On the other hand, limited plant growth increases the share of growth dilution on the overall dissipation.

The major outcome of this study is 114 regression model equations of tested pesticide dissipation of in five vegetable species. These models can be used in practice for monitoring of pesticide residues in products at harvest in dependence on term of application; the knowledge of dissipation rate enables establishing APHI for targeted limits of pesticide residues in product at harvest, e.g. for a limit of 25% MRL or 0.01 mg·kg^−1^. In addition, based on the models, it is possible to differentiate between the pesticides for which prolonging of the PHI is not necessary and those for which some adjustment of PHI is needed. Models of pesticide dissipation and the procedure of APHI calculation can also serve as a basis for expert systems aimed at the regulation of pesticide residues in vegetables. Under these conditions, the key users might be vegetable growers, extension services or retail chains that require vegetable products with pesticide residues lower than official MRLs. Overall, protection of consumer health will occur due to a reduction of pesticide vegetable contamination by residues. The other challenging application of the developed models is their use for registration purposes and updating of respective MRLs and PHIs. However, it remains unclear if reduced pesticide application amounts will remain effective against the relevant target pests. Hence, understanding the efficacy of reduced pesticide application for controlling relevant pest organisms requires further research.

The outcomes of this study might nevertheless help to identify a selection of candidate pesticides for stricter pesticide legislation, specifically in the case of pesticides with a higher incidence of residues in food crops, longer half-lives and longer APHI_0.01_. Considering the focus of future research in this field, the monitoring of levels of rapid dissipation of active substances from the first day after application would be useful as it provides important data for comparison of initial deposits in particular crops with the literature data (18). Evaluation of relationships between MRL, PHI, and acute reference doses (ARfDs) is a challenge for further research of pesticide residues in products. Finally, the models characterizing pesticide dissipation can also be used for screening residue-related consumer exposure, thus informing related risk assessments.

## Figures and Tables

**Table 1:** Crops, planting dates, varieties, as well as the dates of pesticide application and crop harvest in semi-field experiments.

Crop	Planting Date	Variety	Application Date			Harvest Date			
Lettuce ^a^	6 July 2011	Diamantinus	2 August	11 August	16 August	19 August	23 August	29 August	2 September
Onion ^b^	4 April 2012	Wellington	7 August	13 August	17 August	20 August	27 August	*	14 September
Onion ^b^	17 April 2013	Wellington	6 August	12 August	16 August	19 August	26 August	2 September	9 September
Leek ^a^	19 June 12008	Prelina	30 September	6 October	10 October	13 October	20 October	29 October	3 November
Leek ^a^	20 April 2009	Bandit	21 September	28 September	2 October	5 October	12 October	19 October	26 October
Carrot ^a^	15 April 2009	Nerac F1	21 September	28 September	2 October	5 October	10 October	19 October	26 October
Parsley ^a^	18 April 2010	Eagle	3 September	10 September	13 September	16 September	23 September	29 September	7 October

a: semi-field experiments at the Crop Research Institute; b: semi-field experiments at the Czech University of Life Sciences Prague, *: crops not harvested in this term.

**Table 2:** Pesticide residues in iceberg lettuce modelled in terms of the corresponding pre-harvest interval (PHI) according to a list of registered products ([25] the action pre-harvest interval (APHI) was calculated for 25% maximum residue limit (MRL)(APHI_25_)) and a 0.01 mg kg^−1^ limit (APHI_0.01_). The MRL is cited according to the EU Pesticides Database [24].

Active Substance	MRL	Model	PHI	APHI_25_	Suggested PHI for APHI_25_	APHI_0.01_	Suggested PHI for APHI_0.01_
	(mg kg^−1^)	(mg kg^−1^)	(Days)	(Days) ^e^		(Days) ^e^	
Acetamiprid	3	0.0342	3–7 ^b^	0	7	13.2	
Azoxystrobin	15	0.0006	14	2.7	14	13.6	14
Beta-Cyfluthrin	1	0.0005	7 ^b^	0	7	2.9	7
Cypermethrin	2	0.0012	14 ^b^	2.9	14	13.2	14
Deltamethrin	0.5	0.0001	7–14 ^b^	0.5	14	6.7	14
Difenoconazole	4	0.0007	3–14 ^b^	2.1	14	12.5	14
Dimethoate	0.01 ^a^	0.0006	21	21.4 ^d^	21	14.7	21
Dimethomorph	15	<0.0001	21	4.3	21	11.9	21
Chlorpyrifos	0.01 ^a^	0.0044	14 ^b^	20.2 ^d^		16.4	
Indoxacarb	3	0.0028	14	0	14	13.5	14
Iprodione	0.01 ^a,f^	0.0045	14	20.3 ^d^		16.5	
Lambda-Cyhalothrin	0.15	0.0093	7	4.7	7	9.1	
Mandipropamid	25	0.0067	3–14 ^b^	0	14	17.4	
Metalaxyl-M	3	0.0004	14	0.1	14	10.6	14
Methoxyfenozide	4	0.0002	14 ^c^	4.3	14	12.2	14
Pirimicarb	1.5	0.0095	7	0	7	9.2	
Pymetrozine	3	0.0217	7	2.9	7	10.7	
Spinosad	10	0.0001	14	0	14	8.6	14
Tebuconazole	0.5	0.1449	7 ^c^	9.7		16.1	
Thiacloprid	1	0.0398	7	5.6	7	12.1	
Thiamethoxam	5	0.0056	3 ^b^	0	3	1.0	3

^a^ Limit corresponds to the practical limit of quantification (LOQ) of the analytical method; ^b^ PHI listed for pesticide application to another vegetable; for the APHI25 and APHI0.01 calculation, a longer PHI was used; ^c^ Currently only allowed on fruit trees (methoxyfenozide, tebuconazole) or cereals (fluoxastrobin, prothioconazole); ^d^ APHI25 is longer than APHI0.01; in this case, APHI0.01 (zero-residue production) must be used instead of APHI25; ^e^ In cases when the calculated APHI was shorter than the PHI, the recommended PHI should be followed by farmers; ^f^ Iprodione: MRL applicable from 31 July 2019 [26].

**Table 3:** Pesticide residues in onion.

Active Substance	MRL	Model	PHI	APHI_25_	Suggested PHI for APHI_25_	APHI_0.01_	Suggested PHI for APHI_0.01_
	(mg kg^−1^)	(mg kg^−1^)	(Days)	(Days) ^e^		(Days) ^e^	
Abamectin 2012–13	0.01 ^a^	x	3-7 ^b^		7		7
Acetamiprid 2012	0.02 ^a^	0.0013	3-7 ^b^	2.3 ^d^	7	0	7
Acetamiprid 2013	0.02 ^a^	0.0081	3-7 ^b^	13.1 ^d^		7.7	
Acetamiprid 2012–13	0.02 ^a^	0.0046	3–7 ^b^	8.8 ^d^		4.2	7
Azoxystrobin 2012	10	0.0040	14	0	14	12.7	14
Azoxystrobin 2013	10	0.0093	14	0	14	18.3	
Azoxystrobin 2012–13	10	0.0067	14	0	14	16.6	
Boscalid 2012	5	0.0360	14	0	14	41.4	
Boscalid 2013	5	0.0673 ^n.s.^	14		14		14
Chlorantraniliprole 2013	0.01 ^a^	0.0001	14^b^	8.6^d^	14	3.7	14
Cymoxanil 2012	0.01 ^a^	<0.0001	14^b^	4.2^d^	14	3.2	14
Cymoxanil 2013	0.01 ^a^	x	14^b^		14		14
Cypermethrin 2012	0.1	0.0013	14^b^	0	14	0	14
Cypermethrin 2013	0.1	0.0031 ^n.s.^	14^b^		14		14
Cyprodinil 2012	0.3	0.0061	14	0	14	14.1	
Cyprodinil 2013	0.3	0.0051	14	7.9	14	16.0	
Cyprodinil 2012–13	0.3	0.0054	14	6.0	14	15.7	
Deltamethrin 2012–13	0.06	x	10		10		10
Difenoconazole 2012	0.5	0.0006	3–14 ^b^	0	14	0	14
Difenoconazole 2013	0.5	0.0023	3–14 ^b^	0	14	0	14
Dimethoate 2012	0.01 ^a^	0.0005	14	10.5^d^	14	3.2	14
Dimethoate 2013	0.01 ^a^	0.0007 ^n.s.^	14		14		14
Dimethomorph 2012	0.6	0.0011	14	0	14	7.3	14
Dimethomorph 2013	0.6	0.0028	14	2.8	14	13.6	14
Dimethomorph 2012–13	0.6	0.0019	14	0.5	14	11.7	14
Fludioxonil 2012	0.5	0.0004	14	0	14	8.2	14
Fludioxonil 2013	0.5	0.0183	14	5.1	14	22.9	
Fluoxastrobin 2012	0.04	0.0002	35 ^c^	2.3	35	2.3	35
Fluoxastrobin 2013	0.04	0.0007	35 ^c^	10.5	35	10.5	35
Fluoxastrobin 2012–13	0.04	0.0006	35 ^c^	6.6	35	6.6	35
Chlorpyrifos 2012	0.2	0.0018	14 ^b^	0	14	0	14
Chlorpyrifos 2013	0.2	0.0020	14 ^b^	0	14	4.7	14
Chlorpyrifos 2012–13	0.2	0.0019	14 ^b^	0	14	2.0	14
Indoxacarb 2012	0.02 ^a^	0.0016	1–14 ^b^	3.3 ^d^	14	0	14
Indoxacarb 2013	0.02 ^a^	0.006 ^n.s.^	1–14 ^b^		14		14
Iprodione 2012	0.01 ^a.f^	0.0012	3–28 ^b^	32.9 ^d^		24.3	28
Iprodione 2013	0.01 ^a,f^	0.0108	3–28 ^b^	53.3 ^d^		38.2	
Iprodione 2012–13	0.01 ^a,f^	0.0105	3–28 ^b^	54.7 ^d^		37.9	
Lambda-cyhalothrin 2012–13	0.2	x	7–14 ^b^		14		14
Mandipropamid 2012	0.1	0.0023	3–14 ^b^	2.4	14	8.7	14
Mandipropamid 2013	0.1	0.0052	3–14 ^b^	11.5	14	15.7	
Mandipropamid 2012–13	0.1	0.0039	3–14 ^b^	9.3	14	13.9	14
Metalaxyl-M 2012	0.5	0.0049 ^n.s^^.^	7		7		7
Metalaxyl-M 2013	0.5	0.0083	7	0	7	8.1	
Methoxyfenozide 2012	0.01 ^a^	0.0094	14 ^c^	40.8 ^d^		17.5	
Methoxyfenozide 2013	0.01 ^a^	0.0156	14 ^c^	40.3 ^d^		24.0	
Methoxyfenozide 2012–13	0.01 ^a^	0.0124	14 ^c^	39.9 ^d^		21.5	
Pirimicarb 2012	0.1	0.0005	14	0	14	4.1	14
Pirimicarb 2013	0.1	0.0009	14	7.2	14	10.4	14
Pirimicarb 2012–13	0.1	0.0007	14	5.4	14	8.7	14
Propamocarb-hydrochloride 2013	2	0.2499	7	5.5	7	27.1	
Prothioconazole 2012	0.05	x	35 ^c^		35		35
Prothioconazole 2013	0.05	0.0008	35 ^c^	10.2	35	13.1	35
Pyraclostrobin 2012	1.5	0.0037	14	0	14	8.3	14
Pyraclostrobin 2013	1.5	0.0168 ^n.s.^	14		14		14
Spinosad 2012	0.07	0.0038 ^n.s.^	7		7		7
Spinosad 2013	0.07	0.0050	7	0.7	7	4.6	7
Tebuconazole 2012	0.15	0.0209	7 ^c^	4.2	7	15.8	
Tebuconazole 2013	0.15	0.0471	7 ^c^	12.3		29.1	
Tebuconazole 2012–13	0.15	0.0342	7 ^c^	8.3		23.3	
Thiacloprid 2012	0.01 ^a^	0.0100	21 ^b^	9.2 ^d^	21	0.8	21
Thiacloprid 2013	0.01 ^a^	0.0003	21 ^b^	15.9 ^d^	21	11.1	21
Thiacloprid 2012–13	0.01 ^a^	0.0006	21 ^b^	14.5 ^d^	21	9.3	21
Thiamethoxam 2012	0.01 ^a^	x	3 ^b^		3		3
Thiamethoxam 2013	0.01 ^a^	0.0040	3 ^b^	4.4 ^d^	3	3.2	

× the model was not established due to the rapid dissipation of the active substance in the crop; ^n.s.^: non-significant model (*R*^2^ < 0.5); ^a^ Limit corresponds to the practical limit of quantification (LOQ) of the analytical method; ^b^ PHI listed for pesticide application to another vegetable; for the APHI25 and APHI0.01 calculation, a longer PHI was used; ^c^ Currently only allowed on fruit trees (methoxyfenozide, tebuconazole) or cereals (fluoxastrobin, prothioconazole); ^d^ APHI25 is longer than APHI0.01; in this case, APHI0.01 (zero-residue production) must be used instead of APHI25; ^e^ In cases when the calculated APHI was shorter than the PHI, the recommended PHI should be followed by farmers; ^f^ Iprodione: MRL applicable from 31 July 2019 [26].

**Table 4:** Pesticide residues in leek.

Active Substance	MRL	Model	PHI	APHI_25_	Suggested PHI for APHI_25_	APHI_0.01_	Suggested PHI for APHI_0.01_
	(mg kg^−1^)	(mg kg^−1^)	(Days)	(Days) ^e^		(Days) ^e^	
Abamectin 2009	0.01 ^a^	x	3–7 ^b^		7		7
Acetamiprid 2008	0.01 ^a^	0.0152	3–7 ^b^	30.1 ^d^		14.1	
Acetamiprid 2009	0.01 ^a^	0.0130 ^n.s.^	3–7 ^b^		7		7
Azoxystrobin 2008	10	0.0969	21	0	21	54.8	
Azoxystrobin 2009	10	0.0247	21	0	21	33.7	
Azoxystrobin 2008–09	10	0.0636	21	0	21	44.7	
Cypermethrin Nurelle D 2008	0.5	0.0431 ^n.s.^	14 ^b^		14		14
Cypermethrin Vaztak 2009	0.5	0.0413	7–14 ^b^	0	14	50.1	
Deltamethrin 2008	0.3	0.0250	10	0	10	39.2	
Deltamethrin 2009	0.3	0.0129	10	0	10	15.5	
Difenoconazole 2009	0.6	0.0508	3–14 ^b^	8.5	14	33.9	
Chlorpyrifos 2008	0.01 ^a^	0.0484	14 ^b^	50.9 ^d^		35.8	
Lambda-Cyhalothrin 2008	0.07	0.0070 ^n.s.^	7–14 ^b^		14		14
Lambda-Cyhalothrin 2009	0.07	0.0107	7–14 ^b^	9.8	14	19.8	
Pyridaben 2008	0.05 ^a^	0.0241	21 ^g^	38.6		42.2	
Pyridaben 2009	0.05 ^a^	0.0647 ^n.s.^	21 ^g^		21		21
Spinosad 2009	0.2	0.0489	7	9.1		25.3	
Tebuconazole 2009	0.6	0.3535	7 ^c^	15.2		33.8	
Thiacloprid 2008	0.1	0.0233	3–21 ^b^	27.1		38.8	
Thiacloprid 2009	0.1	0.0006	3–21 ^b^	12.1	21	15.9	21
Thiacloprid 2008–09	0.1	0.0099	3–21 ^b^	20.0	21	27.9	
Thiamethoxam 2008	0.01 ^a^	0.0727	3 ^b^	43.6 ^d^		27.3	
Thiamethoxam 2009	0.01 ^a^	0.0221 ^n.s.^	3 ^b^		3		3

× the model was not established due to the rapid dissipation of the active substance in the crop; ^n.s.^: non-significant model (*R*^2^ < 0.5); ^a^ Limit corresponds to the practical limit of quantification (LOQ) of the analytical method; ^b^ PHI listed for pesticide application to another vegetable; for the APHI25 and APHI0.01 calculation, a longer PHI was used; ^c^ Currently only allowed on fruit trees (methoxyfenozide, tebuconazole) or cereals (fluoxastrobin, prothioconazole); ^d^ APHI25 is longer than APHI0.01; in this case, APHI0.01 (zero-residue production) must be used instead of APHI25; ^e^ In cases when the calculated APHI was shorter than the PHI, the recommended PHI should be followed by farmers; ^g^: currently only allowed on ornamentals.

**Table 5:** Pesticide residues in carrot.

Active Substance Root (R)/Leaves (L)	MRL	Model	PHI	APHI_25_	Suggested PHI for APHI_25_	APHI_0.01_	Suggested PHI for APHI_0.01_
	(mg kg^−1^)	(mg kg^−1^)	(Days)	(Days) ^e^		(Days) ^e^	
Acetamiprid (R)	0.01 ^a^	0.0001	7 ^b^	5.3 ^d^	7	3.8	7
Acetamiprid (L)	3	0.3350	7 ^b^	3.8	7	33.2	
Cypermethrin (R)	0.05 ^a^	0.0026	14 ^b^	3.3	14	5.5	14
Cypermethrin (L)	2	0.5593	14 ^b^	21.6		124.8	
Azoxystrobin (R)	1	0.0177	14	5.2	14	21.6	
Azoxystrobin (L)	70	2.1616	14	4.8	14	54.3	
Deltamethrin (R)	0.02 ^a^	x	14 ^b^		14		14
Deltamethrin (L)	2	0.1072	14 ^b^	0	14	57.8	
Difenoconazole (R)	0.4	0.0208 ^n.s.^	14 ^b^		14		14
Difenoconazole (L)	10	0.6157	14 ^b^	0	14	74.8	
Lambda-Cyhalothrin (R)	0.04	x	14 ^b^		14		14
Lambda-Cyhalothrin (L)	0.7	0.0808	14 ^b^	8.7	14	45.6	
Spinosad (R)	0.02 ^a^	0.0013	14 ^b^	12.0 ^d^	14	8.6	14
Spinosad (L)	60	0.0333	14 ^b^	0	14	23.6	
Tebuconazole (R)	0.4	0.0764 ^n.s.^	14 ^c^		14		14
Tebuconazole (L)	2	4.4736	14 ^c^	42.1		100.7	
Thiacloprid (R)	0.05	0.0106	14	8.2	14	9.7	14
Thiacloprid (L)	5	0.9069	14	6.3	14	51.7	

× the model was not established due to the rapid dissipation of the active substance in the crop; ^n.s.^: non-significant model (*R*^2^ < 0.5); ^a^ Limit corresponds to the practical limit of quantification (LOQ) of the analytical method; ^b^ PHI listed for pesticide application to another vegetable; for the APHI25 and APHI0.01 calculation, a longer PHI was used; ^c^ Currently only allowed on fruit trees (methoxyfenozide, tebuconazole) or cereals (fluoxastrobin, prothioconazole); ^d^ APHI25 is longer than APHI0.01; in this case, APHI0.01 (zero-residue production) must be used instead of APHI25; ^e^ In cases when the calculated APHI was shorter than the PHI, the recommended PHI should be followed by farmers; Root (R); Leaves (L).

**Table 6:** Pesticide residues in parsley.

Active Substance	MRL	Model	PHI	APHI_25_	Suggested PHI for APHI_25_	APHI_0.01_	Suggested PHI for APHI_0.01_
	(mg kg^−1^)	(mg kg^−1^)	(Days)	(Days) ^e^		(Days) ^e^	
Azoxystrobin (R)	1	0.0272	14	0	14	32.9	
Azoxystrobin (L)	70	0.0029	14	2.5	14	16.4	
Cypermethrin (R)	0.05 ^a^	x	14 ^b^		14		14
Cypermethrin (L)	2	0.1573	14 ^b^	0	14	66.2	
Deltamethrin (R)	0.02 ^a^	x	14 ^b^		14		14
Deltamethrin (L)	2	0.0701	14 ^b^	0	14	51.3	
Difenoconazole (R)	0.4	0.0490 ^n.s.^	14 ^b^		14		14
Difenoconazole (L)	10	0.7473 ^n.s.^	14 ^b^		14		14
Lambda-Cyhalothrin (R)	0.04	x	14 ^b^		14		14
Lambda-Cyhalothrin (L)	0.7	0.0638	14 ^b^	0	14	57.1	
Metalaxyl-M (R)	0.01 ^a^	x	14 ^b^		14		14
Metalaxyl-M (L)	3	0.0007	14 ^b^	0	14	11.3	14
Pirimicarb (R)	0.05	x	7		7		7
Pirimicarb (L)	3	0.4201	7	2.0	7	56.5	
Spinosad (R)	0.02 ^a^	0.0084 ^n.s.^	14 ^b^		14		14
Spinosad (L)	60	0.0861	14 ^b^	0	14	37.2	
Tebuconazole (R)	0.4	0.0490	7 ^c^	0	7	42.6	
Tebuconazole (L)	2	2.2325 ^n.s.^	7 ^c^		7		7
Thiacloprid (R)	0.05	0.0086	7	4.5	7	7.4	
Thiacloprid (L)	5	0.3081 ^n.s.^	7		7		7

× the model was not established due to the rapid dissipation of the active substance in the crop; ^n.s.^: non-significant model (*R*^2^ < 0.5); ^a^ Limit corresponds to the practical limit of quantification (LOQ) of the analytical method; ^b^ PHI listed for pesticide application to another vegetable; for the APHI25 and APHI0.01 calculation, a longer PHI was used; ^c^ Currently only allowed on fruit trees (methoxyfenozide, tebuconazole) or cereals (fluoxastrobin, prothioconazole); ^e^ In cases when the calculated APHI was shorter than the PHI, the recommended PHI should be followed by farmers; Root (R); Leaves (L) (only in carrot and parsley).

## Supplementary Materials

The following are available online at https://www.mdpi.com/2304-8158/9/5/680/s1, Table S1: Overview of active substances, pesticides, and application rates in crops in semi-field experiments for pesticide residue analyses, Table S2: Optimized MS/MS transition parameters of the LC-based method, Table S3: Ions (*m*/*z*) monitored by the GC-MS analytical method, Table S4: Performance characteristics of the validated analytical method (broccoli spiked at 0.1 mg kg^−1^ and 0.01 mg kg^−1^, six replicates), Table S5: Parameters of pesticide residue dissipation models including dissipation half-lives of pesticides in A) iceberg lettuce; B) onion; C) leek; D) carrot; E) parsley, Table S6: The list of active substances of pesticides presented in Figure 1, Table S7: Dissipation half-lives (t_1/2_) of pesticide residues in plants compared with predicted geometric means of the dissipation half-lives (t1_1/2 ref,i_) at 20 °C and the corrected t_1/2 plant, active subst._ according to the Model II, resp. Model III [14], Table S8: The highest detected concentrations of pesticide residues in tested vegetables three to seven days after final treatment.
