# Supplementary material for: Evaluation of Pesticide Residue Dynamics in Lettuce, Onion, Leek, Carrot and Parsley
Source: Foods. 2020 May 25;9(5):680. doi: 10.3390/foods9050680 (PMC7278720; doi:10.3390/foods9050680)
Supplement: Supplementary file 1 [file foods-09-00680-s001.pdf]

Article

Evaluation of Pesticide Residue Dynamics in Lettuce, Onion, Leek, Carrot and Parsley

Tereza Horská [1], František Kocourek [1], Jitka Stará [1], Kamil Holý [1], Petr Mráz [2], František Krátký [2], Vladimír Kocourek [2] and Jana Hajšlová [2,*]

[1] Crop Research Institute, Division of Crop Protection and Plant Health, Drnovska 507, 161 06 Prague, Czech Republic; tereza.horska@vurv.cz (T.H.); kocourek@vurv.cz (F.K.); stara@vurv.cz (J.S.); holy@vurv.cz (K.H.)

[2] Department of Food Analysis and Nutrition, University of Chemistry and Technology, Technicka 3, 166 28 Prague, Czech Republic; petr.mraz@vscht.cz (P.M.); frantisek.kratky@vscht.cz (F.K.); vladimir.kocourek@vscht.cz (V.K.)

* Correspondence: jana.hajslova@vscht.cz

Received: 8 April 2020; Accepted: 20 May 2020; Published: 25 May 2020

Table S1: Overview of active substances, pesticide preparations and application rates in crops in semi-field experiments aimed at monitoring of pesticide residues. x = the application of the pesticide preparations on the crop. (Category: F = fungicide, I = insecticide)

Active substance (category)	Pesticide	Application rate of pesticide		Lettuce 2011	Onion 2012-2013	Leek 2008-2009	Carrot 2009	Parsley 2010
		L or kg ha^{-1}	g ha^{-1}					
Abamectin (I)	Vertimec 1,8 EC	0.1 L	1.8		x[1]			
Abamectin (I)	Vertimec 1,8 EC	0.5 L	9			2009[*, 3]		
Acetamiprid (I)	Mospilan 20 SP	0.12 kg	24			x[3]	x[2]	
Acetamiprid (I)	Mospilan 20 SP	0.15 kg	30		x[2]			
Acetamiprid (I)	Mospilan 20 SP	0.25 kg	50	x[1]				
Azoxystrobin (F)	Ortiva	1 L	250	x[1]	x[3]	x[2]	x[2]	x[1]
Beta-cyfluthrin (I)	Bulldock 25 EC	0.3 L	7.74	x[2]				
Boscalid (F) +<br>Pyraclostrobin (F)	Signum	1.5 kg	400.5<br>100.5		x[1]			
Chlorantraniliprole (I)	Coragen 20 SC	0.06 L	12		2013[*, 3]			
Cymoxanil (F)	Curzate M WG	2.5 kg	112.5		x[2]			
Cypermethrin (I)	Vaztak 10 SC	0.2 L	20			2009[*, 2]	x[2]	x[1]
Cypermethrin (I) +<br>Chlorpyrifos (I)	Nurelle D	0.6 L	30<br>300	x[1]	x[2]	2008[*, 2]		
Cyprodinil (F) +<br>Fludioxonil (F)	Switch	1 kg	375<br>250		x[3]			
Deltamethrin (I)	Decis flow 2,5	0.2 L	5				x[1]	
Deltamethrin (I)	Decis flow 2,5	0.3 L	7.5			x[1]		
Deltamethrin (I)	Decis Mega	0.15 L	7.5	x[2]	x[1]			
Deltamethrin (I)	Decis Mega	0.2 L	10					x[3]
Difenoconazole (F)	Score 250 EC	0.2 L	50	x[2]		2009[*, 1]	x[3]	
Difenoconazole (F)	Score 250 EC	0.4 L	100		x[2]			x[2]
Dimethoate (I)	Bi-58 EC Nové	0.25 L	100	x[3]				
Dimethoate (I)	Bi-58 EC Nové	0.6 L	240		x[2]			

Dimethomorph (F)	Acrobat MZ	2 kg	180	x[1]	x[3]			
Fluoxastrobin (F) + Prothioconazole (F)	Fandango 200 EC	1.25 L	125 125		x[1]			
Indoxacarb (I)	Steward	0.085 kg	25.5	x[3]	x[1]			
Iprodione (F)	Rovral Flo	1 L	255	x[3]	x[2]			
Lambda-cyhalothrin (I)	Karate se Zeon technologií 5 CS	0.2 L	10			x[2]	x[1]	
Lambda-cyhalothrin (I)	Karate se Zeon technologií 5 CS	0.15 L	7.5	x[1]	x[3]			x[3]
Mandipropamide (F)	Revus	0.6 L	150	x[3]	x[3]			
Metalaxyl-M (F)	Ridomil Gold MZ pepite	2 kg	80	x[2]				x[1]
Metalaxyl-M (F)	Ridomil Gold MZ pepite	2.5 kg	100		x[1]			
Methoxyfenozide (I)	Integro	0.4 L	96	x[1]				
Methoxyfenozide (I)	Integro	0.5 L	120		x[2]			
Pirimicarb (I)	Pirimor 50 WG	0.25 kg	125	x[3]				
Pirimicarb (I)	Pirimor 50 WG	0.3 kg	150					x[1]
Pirimicarb (I)	Pirimor 50 WG	0.5 kg	250		x[3]			
Propamocarb-hydrochloride (F)	Infinito	1.6 L	1000		2013[*, 1]			
Pymetrozine (I)	Chess 50 WG	0.4 kg	200	x[3]				
Pyridaben (I)	Sanmite 20 WP	0.375 kg	75			x[3]		
Spinosad (I)	Spintor	0.4 L	96	x[3]				
Spinosad (I)	Spintor	0.5 L	120			2009[*, 2]	x[3]	x[2]
Spinosad (I)	Spintor	0.6 L	144		x[1]			
Tebuconazole (F)	Horizon 250 EW	0.75 L	187.5	x[1]		2009[*, 1]	x[1]	x[3]
Tebuconazole (F)	Horizon 250 EW	1 L	250		x[1]			
Thiacloprid (I)	Calypso 480 SC	0.12 L	57.6					x[2]
Thiacloprid (I)	Calypso 480 SC	0.15 L	72			x[1]	x[3]	
Thiacloprid (I)	Calypso 480 SC	0.2 L	96	x[2]	x[3]			
Thiamethoxam (I)	Actara 25 WG	0.08 kg	20	x[3]	x[2]			
Thiamethoxam (I)	Actara 25 WG	0.15 kg	37.5			x[3]		

*Sprayed in one year only; [1, 2, 3] pesticide mixtures groups for each crop.

Table S2: Optimised MS/MS transition parameters of the LC-based method.

Pesticide	Primary Transition	Cone (V)	Collision (V)	Secondary Transition	Cone (V)	Collision (V)
Avermectin B1a	890.6 > 305.2	20	25	890.6 > 567.4	20	13
Avermectin B1b	875.7 > 145.3	65	25	875.7 > 290.5	20	13
Acetamiprid	223.1 > 126	31	20	223.1 > 56.1	31	14
Azoxystrobin	404.1 > 372	22	15	404.1 > 344.1	22	24
Boscalid	342.9 > 307	30	20	342.9 > 139.9	30	20
Chlorpyrifos	349.9 > 197.9	26	17	351.9 > 199.9	26	17
Chlorantraniliprole	483.9 > 452.93	4	22	483.9 > 285.91	4	14
Clothianidin (metabolite of thiamethoxam)	250 > 169	22	13	250 > 131.9	22	16

Cymoxanil	199 > 128	14	8	199 > 111	14	18
Cypermethrin	433 > 191	17	15	433 > 91.1	17	46
Cyprodinil	226.01 > 93	40	33	226.01 > 108	40	30
Deltametrin	522.9 > 280.9	22	16	522.9 > 93.1	22	46
Desmethyl-pirimicarb (metabolite of pirimicarb)	225.3 > 72	29	19	225.3 > 168.1	29	14
Difenoconazole	406 > 251	37	26	408 > 253	37	27
Dimethoate	230 > 198.9	21	10	230 > 124.9	21	22
Dimethomorph	388.1 > 301.1	36	21	388.1 > 165	36	32
Fludioxonil (ESI$^-$)	247 > 180	42	28	247 > 126	42	35
Fluoxastrobin	459 > 427	27	18	459 > 188	27	36
Indoxacarb	528 > 203.1	26	43	528 > 249.1	26	16
Mandipropamide	412.1 > 328.04	10	14	412.1 > 356.05	10	8
Metalaxyl	280.2 > 220.1	24	15	280.2 > 192.1	24	19
Methoxyfenozide	369.2 > 149	16	16	369.2 > 313.1	16	8
Omethoate (metabolite of dimethoate)	214 > 124.9	23	21	214 > 182.9	23	11
Pirimicarb	239.1 > 72	32	23	239.1 > 182.1	32	16
Propamocarb	189.1 > 102	22	17	189.1 > 144	22	12
Prothioconazole	314.1 > 69,9	44	18	312.03 > 69.9	44	18
Pymetrozine	218.2>105	31	20	218.2 > 78	31	20
Pyraclostrobin	388.11 > 193.9	20	12	388.11 > 163	20	25
Pyridaben	365.1 > 147.1	28	26	365.1 > 309.1	28	12
Spinosad (spinosyn A)	732.5 > 142.1	48	29	732.5 > 98.1	48	63
Spinosad (spinosyn D)	746.5 > 142	48	29	746.5 > 97.9	48	63
Tebuconazole	308.2 > 70	36	20	308.2 > 125	36	38
Thiacloprid	253.1 > 126	34	25	253.1 > 90	34	37
Thiamethoxam	292 > 181.1	22	23	292 > 132	22	24

Table S3: Ions (*m*/*z*) monitored by the GC-MS analytical method

**Analyte**	**Quantitation Ions (*m*/*z*)**	**Confirmation Ions (*m*/*z*)**
Beta-cyfluthrin	163	206, 226
Iprodione	314	316, 245, 187
Lambda-cyhalothrin	181	197, 208

Table S4: Performance characteristics of the validated analytical method (broccoli spiked at 0.1 mg kg^{-1} and 0.01 mg kg^{-1}, six replicates)

**Analyte**	**Concentration at 0.1 mg kg^{-1}**		**Concentration at 0.01 mg kg^{-1}**		**LOQ (mg kg^{-1})**
	**Recovery (%)**	**Repeatability (RSD, %)**	**Recovery (%)**	**Repeatability (RSD, %)**	
Abamectin	106	18	117	20	0.004
Acetamiprid	89	2	98	4	0.002
Azoxystrobin	90	3	95	4	0.002
Beta-cyfluthrin	102	2	116	17	0.01
Boscalid	91	4	82	9	0.002

Chlorpyrifos	91	2	93	8	0.002
Chlorantraniliprole	96	12	72	7	0.002
Cymoxanil	79	5	87	6	0.002
Cypermethrin	90	3	93	11	0.005
Cyprodinil	84	8	87	14	0.002
Deltamethrin	87	5	90	10	0.002
Difenoconazole	90	2	95	4	0.002
Dimethoate	89	4	91	7	0.002
Dimethomorph	89	1	92	4	0.002
Fludioxonil	85	2	82	2	0.002
Fluoxastrobin	90	5	86	3	0.002
Indoxacarb	92	2	98	8	0.002
Iprodione	107	3	-	-	0.03
Lambda-cyhalothrin	99	1	88	9	0.01
Mandipropamide	86	6	88	7	0.002
Metalaxyl	92	3	92	6	0.002
Methoxyfenzide	94	2	94	3	0.002
Pirimicarb	94	6	95	3	0.002
Propamocarb-hydrochloride	81	7	92	7	0.002
Prothioconazole	88	5	85	12	0.004
Pymetrozine	90	7	86	10	0.005
Pyraclostrobin	90	5	88	12	0.002
Pyridaben	92	2	92	4	0.002
Spinosad	90	2	97	2	0.002
Tebuconazole	95	2	91	3	0.002
Thiacloprid	90	3	98	7	0.002
Thiamethoxam	91	3	104	6	0.002

Table **S5A:** Parameters of pesticide residue dissipation models including dissipation half-lives of pesticides in iceberg lettuce. C_0 and k = parameters in the model equation; R^2 = coefficient of determination; df = degrees of freedom; $t_{1/2}$ dissipation half-life

**Active substance**	**C_0**	**k**	**R^2**	**df**	**$t_{1/2}$ (days)**
Acetamiprid	0.655	-0.422	0.996	10	1.64
Azoxystrobin	16.631	-0.725	0.998	10	0.96
Beta-cyfluthrin	0.038	-0.609	0.991	10	1.14
Cypermethrin	1.506	-0.507	0.981	10	1.37
Deltamethrin	0.151	-0.539	0.992	10	1.29
Difenoconazole	2.513	-0.589	0.997	10	1.18
Dimethoate	0.213	-0.277	0.868	10	2.50
Dimethomorph	13.382	-0.808	1.000	10	0.86
Chlorpyrifos	4.257	-0.492	0.993	10	1.41
Indoxacarb	0.281	-0.329	0.980	10	2.11
Iprodione	4.795	-0.498	0.999	10	1.39
Lambda-cyhalothrin	0.157	-0.404	0.896	10	1.72
Mandipropamid	2.350	-0.419	0.996	10	1.66
Metalaxyl-M	0.772	-0.548	0.996	10	1.26

Methoxyfenozide	12.592	-0.782	0.999	10	0.89
Pirimicarb	0.260	-0.472	0.991	10	1.47
Pymetrozine	3.578	-0.729	0.999	6	0.95
Spinosad	0.537	-0.614	0.997	10	1.13
Tebuconazole	5.649	-0.523	0.998	10	1.32
Thiacloprid	4.033	-0.660	0.998	10	1.05
Thiamethoxam	0.012	-0.261	0.761	10	2.65

Table S5B: Parameters of pesticide residue dissipation models including dissipation half-lives of pesticides in onion. x – model was not established due to the rapid dissipation of the active substance in the crop; n.s. – non-significant model (R^2 < 0.500); C0 and k = parameters in the model equation; R^2 = coefficient of determination; df = degrees of freedom; $t_{1/2}$ dissipation half-life

**Active substance**	C_0	k	R^2	**df**	$t_{1/2}$ **(days)**
Abamectin 2012-13	x	x	x	18	
Acetamiprid 2012	0.008	-0.256	0.712	7	2.70
Acetamiprid 2013	0.027	-0.171	0.853	9	4.05
Acetamiprid 2012-13	0.019	-0.200	0.555	18	3.47
Azoxystrobin 2012	0.070	-0.204	0.999	7	3.39
Azoxystrobin 2013	0.387	-0.266	0.978	9	2.61
Azoxystrobin 2012-13	0.225	-0.251	0.623	18	2.76
Boscalid 2012	0.103	-0.075	0.699	7	9.24
Boscalid 2013	0.123	-0.043	0.353 n.s.	9	
Chlorantraniliprole 2013	0.029	-0.379	0.841	9	1.83
Cymoxanil 2012	0.983	-1.909	1.000	7	0.36
Cymoxanil 2013	x	x	x	9	
Cypermethrin 2012	0.006	-0.112	0.585	7	6.17
Cypermethrin 2013	0.005	-0.038	0.041 n.s.	9	
Cyprodinil 2012	0.045	-0.143	0.973	7	4.85
Cyprodinil 2013	0.543	-0.333	0.962	9	2.08
Cyprodinil 2012-13	0.261	-0.277	0.538	18	2.50
Deltamethrin 2012-13	x	x	x	18	
Difenoconazole 2012	0.005	-0.144	0.656	7	4.83
Difenoconazole 2013	0.009	-0.099	0.583	9	6.98
Dimethoate 2012	0.018	-0.254	0.899	7	2.73
Dimethoate 2013	0.003	-0.097	0.178 n.s.	9	
Dimethomorph 2012	0.041	-0.257	0.970	4	2.70
Dimethomorph 2013	0.300	-0.335	0.988	9	2.07
Dimethomorph 2012-13	0.168	-0.320	0.589	15	2.16
Fludioxonil 2012	0.113	-0.396	0.831	7	1.75
Fludioxonil 2013	0.259	-0.189	0.819	9	3.66
Fluoxastrobin 2012	0.012	-0.113	0.674	7	6.16
Fluoxastrobin 2013	0.021	-0.096	0.645	9	7.25
Fluoxastrobin 2012-13	0.016	-0.096	0.536	18	7.20

Chlorpyrifos 2012	0.008	-0.106	0.500	7	6.56
Chlorpyrifos 2013	0.017	-0.155	0.657	9	4.46
Chlorpyrifos 2012-13	0.012	-0.134	0.547	18	5.16
Indoxacarb 2012	0.006	-0.096	0.599	7	7.21
Indoxacarb 2013	0.010	-0.034	0.297 n.s.	9	
Iprodione 2012	0.511	-0.216	0.586	6	3.22
Iprodione 2013	0.333	-0.122	0.813	9	5.66
Iprodione 2012-13	0.225	-0.110	0.586	17	6.32
Lambda-cyhalothrin 2012,13	x	x	x	18	
Mandipropamid 2012	0.036	-0.195	0.979	7	3.56
Mandipropamid 2013	0.300	-0.289	0.962	9	2.40
Mandipropamid 2012-13	0.161	-0.266	0.557	18	2.60
Metalaxyl-M 2012	0.009	-0.090	0.463 n.s.	7	
Metalaxyl-M 2013	0.034	-0.202	0.796	9	3.44
Methoxyfenozide 2012	0.028	-0.079	0.756	7	8.72
Methoxyfenozide 2013	0.076	-0.113	0.794	9	6.14
Methoxyfenozide 2012-13	0.051	-0.101	0.609	18	6.88
Pirimicarb 2012	0.023	-0.277	0.935	7	2.51
Pirimicarb 2013	0.201	-0.386	0.996	9	1.79
Pirimicarb 2012-13	0.110	-0.366	0.609	18	1.89
Propamocarb-hydrochloride 2013	1.358	-0.242	0.926	5	2.87
Prothioconazole 2012	x	x	x	7	
Prothioconazole 2013	0.027	-0.102	0.712	9	6.78
Pyraclostrobin 2012	0.022	-0.127	0.759	7	5.45
Pyraclostrobin 2013	0.028	-0.036	0.241 n.s.	9	
Spinosad 2012	0.004	-0.026	0.067 n.s.	7	
Spinosad 2013	0.019	-0.194	0.663	9	3.58
Tebuconazole 2012	0.061	-0.153	0.855	7	4.53
Tebuconazole 2013	0.098	-0.104	0.650	9	6.64
Tebuconazole 2012-13	0.078	-0.117	0.551	18	5.91
Thiacloprid 2012	0.011	-0.219	0.842	7	3.16
Thiacloprid 2013	0.246	-0.384	0.992	9	1.80
Thiacloprid 2012-13	0.121	-0.356	0.517	18	1.95
Thiamethoxam 2012	x	x	x	7	
Thiamethoxam 2013	0.459	-1.581	1.000	9	0.44

Table S5C: Parameters of pesticide residue dissipation models including dissipation half-lives of pesticides in leek. x – model was not established due to the rapid dissipation of the active substance in the crop; n.s. – non-significant model (R^2 < 0.500); C0 and k = parameters in the model equation; R^2 = coefficient of determination; df = degrees of freedom; $t_{1/2}$ dissipation half-life

**Active substance**	**C_0**	**k**	**R^2**	**df**	**$t_{1/2}$ (days)**
Abamectin 2009	x	x	x	10	
Acetamiprid 2008	0.034	-0.116	0.845	10	5.97
Acetamiprid 2009	0.018	-0.043	0.070 n.s.	10	

Azoxystrobin 2008	1.044	-0.113	0.802	10	6.12
Azoxystrobin 2009	2.172	-0.213	0.888	10	3.25
Azoxystrobin 2008-09	1.427	-0.148	0.793	22	4.68
Cypermethrin Nurelle D 2008	0.132	-0.080	0.480 n.s.	10	
Cypermethrin Vaztak 2009	0.096	-0.060	0.708	10	11.53
Deltamethrin 2008	0.040	-0.047	0.568	10	14.65
Deltamethrin 2009	0.062	-0.157	0.936	10	4.43
Difenoconazole 2009	0.370	-0.142	0.869	10	4.89
Chlorpyrifos 2008	0.269	-0.123	0.914	10	5.66
Lambda-cyhalothrin 2008	0.019	-0.070	0.240 n.s.	10	
Lambda-cyhalothrin 2009	0.030	-0.075	0.760	8	9.27
Pyridaben 2008	0.137	-0.083	0.745	10	8.38
Pyridaben 2009	0.148	-0.039	0.162 n.s.	10	
Spinosad 2009	0.124	-0.132	0.732	10	5.23
Tebuconazole 2009	1.378	-0.194	0.900	10	3.57
Thiacloprid 2008	0.211	-0.105	0.905	10	6.61
Thiacloprid 2009	0.462	-0.321	0.939	10	2.16
Thiacloprid 2008-09	0.253	-0.155	0.822	22	4.49
Thiamethoxam 2008	0.102	-0.113	0.922	10	6.12
Thiamethoxam 2009	0.025	-0.046	0.080 n.s.	10	

Table S5D: Parameters of pesticide residue dissipation models including dissipation half-lives of pesticides in carrot. x – model was not established due to the rapid dissipation of the active substance in the crop; n.s. – non-significant model (R^2 < 0.500); C0 and k = parameters in the model equation; R^2 = coefficient of determination; df = degrees of freedom; $t_{1/2}$ dissipation half-life

**Active substance**	**C_0**	**k**	**R^2**	**df**	**$t_{1/2}$ (days)**
Acetamiprid (R)	0.349	-1.238	1.000	10	0.56
Acetamiprid (L)	1.321	-0.196	0.959	10	3.22
Azoxystrobin (R)	0.687	-0.261	0.960	10	2.65
Azoxystrobin (L)	36.116	-0.201	0.899	10	2.61
Cypermethrin (R)	0.017	-0.135	0.516	10	5.13
Cypermethrin (L)	1.135	-0.051	0.713	10	11.50
Deltamethrin (R)	x	x	x	10	
Deltamethrin (L)	0.333	-0.081	0.695	10	8.89
Difenoconazole (R)	0.027	-0.018	0.072 n.s.	10	
Difenoconazole (L)	2.424	-0.098	0.775	10	7.08
Lambda-cyhalothrin (R)	x	x	x	10	
Lambda-cyhalothrin (L)	0.344	-0.104	0.736	10	6.42
Spinosad (R)	0.057	-0.272	0.920	10	2.55
Spinosad (L)	3.109	-0.324	0.986	10	2.14
Tebuconazole (R)	0.126	-0.071	0.416 n.s.	10	
Tebuconazole (L)	8.348	-0.089	0.783	10	6.95
Thiacloprid (R)	0.040	-0.191	0.855	10	3.63
Thiacloprid (L)	2.449	-0.142	0.961	10	3.98

Notes: root (R); leaves (L)

Table S5E: Parameters of pesticide residue dissipation models including dissipation half-lives of pesticides in parsley. x – model was not established due to the rapid dissipation of the active substance in the crop; n.s. – non-significant model ($R^2 < 0.500$); C0 and k = parameters in the model equation; R^2 = coefficient of determination; df = degrees of freedom; $t_{1/2}$ dissipation half-life

Active substance	C_0	*k*	R^2	df	$t_{1/2}$ (days)
Cypermethrin (R)	x	x	x	10	
Cypermethrin (L)	0.464	-0.077	0.786	10	8.97
Azoxystrobin (R)	0.101	-0.093	0.778	10	7.42
Azoxystrobin (L)	66.654	-0.718	0.985	10	0.97
Deltamethrin (R)	x	x	x	10	
Deltamethrin (L)	0.213	-0.080	0.882	10	8.72
Difenoconazole (R)	0.085	-0.039	0.440 n.s.	7	
Difenoconazole (L)	1.270	-0.038	0.205 n.s.	7	
Lambda-cyhalothrin (R)	x	x	x	10	
Lambda-cyhalothrin (L)	0.157	-0.064	0.707	10	10.89
Metalaxyl-M (R)	x	x	x	6	
Metalaxyl-M (L)	0.568	-0.478	0.937	6	1.45
Pirimicarb (R)	x	x	x	10	
Pirimicarb (L)	0.880	-0.106	0.926	10	6.56
Spinosad (R)	0.022	-0.069	0.396 n.s.	10	
Spinosad (L)	0.755	-0.155	0.781	10	4.47
Tebuconazole (R)	0.076	-0.064	0.600	10	10.88
Tebuconazole (L)	2.778	-0.031	0.422 n.s.	10	
Thiacloprid (R)	0.018	-0.102	0.532	10	6.81
Thiacloprid (L)	0.528	-0.077	0.383 n.s.	10	

root (R); leaves (L).

Table S6: The list of active substances of pesticides presented in Figure 1

	Lettuce	Onion	Leek
**1**	Thiametoxam	Boscalid 2012	Deltamethrin 2008
**2**	Dimethoate	Indoxacarb 2012	Cypermethrin Vaztak 2009
**3**	Indoxacarb	Fluoxastrobin 2012-13	Lambda-cyhalothrin 2009
**4**	Lambda-cyhalothrin	Difenoconazole 2013	Pyridaben 2008
**5**	Mandipropamid	Methoxyfenzide 2012-13	**mean pesticide**
**6**	Acetamiprid	Prothioconazole 2013	Thiametoxam 2008
**7**	Pirimicarb	Iprodione 2012-13	Acetamiprid 2008
**8**	**mean pesticide**	Cypermethrin 2012	Chlorpyrifos 2008
**9**	Chlorpyrifos	Tebuconazole 2012-13	Spinosad 2009
**10**	Iprodione	Pyraclostrobin 2012	Difenoconazole 2009
**11**	Cypermethrin	Chlorpyrifos 2012-13	Azoxystrobin 2008-09
**12**	Tebuconazole	Difenoconazole 2012	Thiacloprid 2008-09
**13**	Deltamethrin	**mean pesticide**	Deltamethrin 2009
**14**	Metalaxyl-M	Fludioxonil	Tebuconazole 2009

**15**	Difenoconazole	Spinosad 2013	×
**16**	Beta-cyfluthrin	Acetamiprid 2012-13	×
**17**	Spinosad	Metalayl-M 2013	×
**18**	Thiacloprid	Propamocarb-hydrochloride 2013	×
**19**	Azoxystrobin	Azoxystrobin 2012-13	×
**20**	Pymetrozine	Dimethoate 2012	×
**21**	Methoxifenzide	Mandipropamid 2012-13	×
**22**	Dimethomorph	Cyprodinil 2012-13	×
**23**	×	Dimethomorph 2012-13	×
**24**	×	Thiacloprid 2012-13	×
**25**	×	Pirimicarb 2012-13	×
**26**	×	Clorantraniliprol 2013	×
**27**	×	Fludioxonil	×
**28**	×	Thiametoxam 2013	×
**29**	×	Cymoxanil 2012	×

Table S6: The list of active substances of pesticides presented in Figure 1 (continue)

	Carrot Root	Carrot Leaves	Parsley Roots	Parsley Leaves
**1**	cypermethrin	×	Tebuconazole	×
**2**	Thiacloprid	×	**mean pesticide**	×
**3**	**mean pesticide**	×	Azoxystrobin	×
**4**	Azoxystrobin	×	Thiacloprid	×
**5**	Spinosad	×	×	×
**6**	Acetamiprid	×	×	×
**7**	×	×	×	×
**8**	×	×	×	×
**9**	×	×	×	×
**10**	×	×	×	×
**11**	×	×	×	×
**12**	×	×	×	×
**13**	×	×	×	×
**14**	×	×	×	×
**15**	×	×	×	×
**16**	×	×	×	×
**17**	×	×	×	×
**18**	×	×	×	×
**19**	×	cypermethrin	×	×
**20**	×	Deltamethrin	×	×
**21**	×	Difenoconazole	×	Lambda-cyhalothrin
**22**	×	Tebuconazole	×	cypermethrin
**23**	×	Lambda-cyhalothrin	×	Deltamethrin
**24**	×	**mean pesticide**	×	Pirimicarb
**25**	×	Thiacloprid	×	**mean pesticide**
**26**	×	Acetamiprid	×	Spinosad

**27**	×	Azoxystrobin	×	Metalayl-M
**28**	×	Spinosad	×	Azoxystrobin
**29**	×	×	×	×

Table S7: Dissipation half-lives ($t_{1/2}$) of pesticide residues in plants compared with predicted geometric means of the dissipation half-lives ($t_{1/2\ \text{ref,i}}$) at 20 °C and the corrected $t_{1/2\ \text{plant, active subst.}}$ according to the Model II, resp. Model III [7]

Active Substance	$t_{1/2}$ (day)	$t_{1/2\ \text{ref,i}}$ (day)	$t_{1/2\ \text{plant, active subst.}}$ (day)
LETTUCE 20 °C *			
Acetamiprid	1.64	5.69	3.67
Azoxystrobin	0.96	3.53	2.28
Beta-cyfluthrin	1.14	2.39	1.54
Cypermethrin	1.37	4.24	2.74
Deltamethrin	1.29	3.76	2.43
Difenoconazole	1.18	5.02	3.24
Dimethoate	2.50	3.61	2.33
Dimethomorph	0.86	5.14	3.32
Chlorpyrifos	1.41	4.01	2.59
Indoxacarb	2.11	2.99	1.93
Iprodione	1.39	6.92	4.47
Lambda-cyhalothrin	1.72	2.86	1.85
Mandipropamide	1.66	3.46	2.23
Metalaxyl-M	1.26	3.74	2.41
Methoxyfenozide	0.89	6.86	4.43
Pirimicarb	1.47	4.55	2.94
Pymetrozine	0.95	2.94	1.90
Spinosad	1.13	4.42	2.85
Tebuconazole	1.32	7.67	4.95
Thiacloprid	1.05	3.76	2.43
Thiamethoxam	2.65	3.97	2.56
ONION 18 °C (18.3 / 17.3) *			
Acetamiprid 2012-13	3.47 (2.70 / 4.05)	5.69	4.42 (4.36 / 4.56)
Azoxystrobin 2012-13	2.76 (3.39 / 2.61)	3.53	2.74 (2.70 / 2.83)
Boscalid 2012	9.24	6.63	5.31
Chlorantraniliprole 2013	1.83	3.38	2.71
Cymoxanil 2012	0.36	1.61	1.23
Cypermethrin 2012	6.17	4.24	3.25
Cyprodinil 2012-13	2.50 (4.85 / 2.08)	5.70	4.42 (4.36 / 4.57)
Difenoconazole 2012-13	– (4.83 / 6.98)	5.02	– (3.84 / 4.02)
Dimethoate 2012	2.73	3.61	2.76
Dimethomorph 2012-13	2.16 (2.70 / 2.07)	5.14	3.99 (3.93 / 4.12)
Fludioxonil 2012-13	– (1.75 / 3.66)	5.24	– (4.01 / 4.20)
Fluoxastrobin 2012-13	7.20 (6.16 / 7.25)	6.14[a]	6.73 (6.64 / 6.95)
Chlorpyrifos 2012-13	5.16 (6.56 / 4.46)	4.01	3.11 (3.07 / 3.21)
Indoxacarb 2012	7.21	2.99	2.29
Iprodione 2012-13	6.32 (3.22 / 5.66)	6.92	5.37 (5.30 / 5.55)
Mandipropamide 2012-13	2.60 (3.56 / 2.40)	3.46	2.69 (2.65 / 2.77)

Metalaxyl-M 2013	3.44	3.74	3.00
Methoxyfenozide 2012-13	6.88 (8.72 / 6.14)	6.86	5.32 (5.25 / 5.50)
Pirimicarb 2012-13	1.89 (2.51 / 1.79)	4.55	3.53 (3.48 / 3.65)
Propamocarb-hydrochloride 2013	2.87	1.10	0.88
Prothioconazole 2013	6.78	7.19 [a]	8.14
Pyraclostrobin 2012	5.45	3.90	2.99
Spinosad 2013	3.58	4.42	3.54
Tebuconazole 2012-13	5.91 (4.53 / 6.64)	7.67	5.95 (5.87 / 6.15)
Thiacloprid 2012-13	1.95 (3.16 / 1.80)	3.76	2.92 (2.88 / 3.01)
Thiamethoxam 2013	0.44	3.97	3.18
LEEK 10 °C (9.0 / 10.5) *			
Acetamiprid 2008	5.97	5.69	9.47
Azoxystrobin 2008-09	4.68 (6.12 / 3.25)	3.53	5.61 (5.87 / 5.48)
Cypermethrin Vaztak 2009	11.53	4.24	6.58
Deltamethrin 2008-2009	– (14.65 / 4.43)	3.53	– (6.26 / 5.84)
Difenoconazole 2009	4.89	5.02	7.80
Chlorpyrifos 2008	5.66	4.01	6.67
Lambda-cyhalothrin 2009	9.27	2.86	4.44
Pyridaben 2008	8.38	4.84	8.05
Spinosad 2009	5.23	4.42	6.86
Tebuconazole 2009	3.57	7.67	11.91
Thiacloprid 2008-09	4.49 (6.61 / 2.16)	3.76	5.97 (6.61 / 5.84)
Thiamethoxam 2008	6.12	3.97	6.60
CARROT 10.5 °C *			
root			
Acetamiprid	0.56	5.69	8.84
Azoxystrobin	2.65	3.53	5.48
Cypermethrin	5.13	4.24	6.58
Spinosad	2.55	4.42	6.86
Thiacloprid	3.63	3.76	5.84
leaves			
Acetamiprid	3.22	5.69	8.84
Azoxystrobin	2.61	3.53	5.48
Cypermethrin	11.50	4.24	6.58
Deltamethrin	8.89	3.76	5.84
Difenoconazole	7.08	5.02	7.80
Lambda-cyhalothrin	6.42	2.86	4.44
Spinosad	2.14	4.42	6.86
Tebuconazole	6.95	7.67	11.91
Thiacloprid	3.98	3.76	5.84
PARSLEY 12 °C *			
root			
Azoxystrobin	7.42	3.53	5.12
Tebuconazole	10.88	7.67	11.12
Thiacloprid	6.81	3.76	5.45
leaves			
Azoxystrobin	0.97	3.53	5.12
Cypermethrin	8.97	4.24	6.15

Deltamethrin	8.72	3.76	5.45
Lambda-cyhalothrin	10.89	2.86	4.15
Metalaxyl-M	1.45	3.74	5.42
Pirimicarb	6.56	4.55	6.59
Spinosad	4.47	4.42	6.41

* The average temperature from the first chemical treatment until the last harvest; [a] predicted half-lives for fluoxastrobin and prothioconazole was calculated using model III and data from PubChem CID=11048796; CID=6451142.

Table S8: The highest detected concentrations of pesticide residues (mg kg^{-1}) in tested vegetables three to seven days after treatment

Active Substance	Lettuce	Onion *	Leek *	Carrot Root	Parsley Root	Carrot Leaves	Parsley Leaves
Azoxystrobin	1.888	0.176	1.180	0.323	0.087	19.32	7.748
Cypermethrin	0.330	0.009	0.116	0.014	0.000	1.122	0.441
Deltamethrin	0.030	0.001	0.042/0.039	0.000	0.000	0.285	0.164
Difenoconazole	0.429	0.003/0.006	0.26	0.055	0.090	1.964	1.478
Lambda-cyhalothrin	0.047	0.000	0.032	0.000	0.000	0.277	0.138
Spinosad	0.085	0.011	0.078	0.025	0.028	1.176	0.452
Tebuconazole	1.176	0.072	0.810	0.159	0.086	7.404	3.010
Thiacloprid	0.557	0.078	0.179	0.023	0.019	1.609	0.437

* the highest detected concentration from two seasons supposing the data from two years were comparable.

© 2020 by the authors. Licensee MDPI, Basel, Switzerland. This article is an open access article distributed under the terms and conditions of the Creative Commons Attribution (CC BY) license (http://creativecommons.org/licenses/by/4.0/).
